# Protective effect of heat-processed *Gynostemma pentaphyllum* on high fat diet-induced glucose metabolic disorders mice

**DOI:** 10.3389/fphar.2023.1215150

**Published:** 2023-09-25

**Authors:** Jin-Bo Xie, Peng Xie, Mei Guo, Fang-Fang Li, Man-Yu Xiao, Yan-Shuang Qi, Wen-Jing Pei, Hao-Tian Luo, Yu-Long Gu, Xiang-Lan Piao

**Affiliations:** School of Pharmacy, Minzu University of China, Beijing, China

**Keywords:** *Gynostemma pentaphyllum*, gypenoside, lipid lowering, glucose metabolic disorders, AGE-RAGE signaling pathway

## Abstract

Glucose metabolic disorders (GMD) can promote insulin resistance (IR) and diabetes, and damage liver and kidney. *Gynostemma pentaphyllum* is commonly used in the clinical treatment of diabetes, but the research on its main active constituents and GMD has not been reported yet. This study explores the therapeutic potential of gypenosides of heat-processed *Gynostemma pentaphyllum* (HGyp) on high-fat diet-induced GMD in mice. HGyp was administered at different doses for 12 weeks. The investigation encompassed an array of parameters, including body weight, blood lipids, blood glucose, and liver tissue components. Metabolomic and network analyses were conducted to uncover potential targets and pathways associated with HGyp treatment. The results revealed that HGyp alleviated GMD by reducing body weight, blood glucose, and improving blood lipids levels, while increasing liver glycogen and antioxidant enzyme levels. Additionally, HGyp exhibited protective effects on liver and kidney health by reducing tissue damage. Fourteen blood components were detected by LC-MS. Metabolomic and network analyses indicated the potential engagement of the AGE-RAGE signaling pathway in the therapeutic effects of HGyp.Furthermore, Western blot and ELISA assays confirmed that HGyp upregulated GLO1 and GLUT4 while down-regulating AGEs and RAGE expression in liver tissue. In light of these findings, HGyp demonstrates promise as a potential therapeutic candidate for combating GMD, warranting further exploration in the development of therapeutic strategies or functional products.

## 1 Introduction

Glycolipidemic metabolic diseases include chronic diseases such as abnormal blood glucose, abnormal blood lipid, obesity, diabetes, nonalcoholic fatty liver disease, and their incidence rate remains high. The latest data shows that the global adult obesity rate is 12% (Afshin et al., 2017), and the prevalence of diabetes is 9.3% (Aschner et al., 2021). Their common feature is glucose metabolism disorder (GMD) and lipid metabolism disorder, which causes damage to multiple organs throughout the body. Lipid metabolism disorder refers to various disorders including total cholesterol (TC), triglyceride (TG), low density lipoprotein cholesterol (LDL-C), high-density lipoprotein cholesterol (HDL-C), etc., (Zhang Y. et al., 2018). GMD can be divided into hypoglycemia and hyperglycemia. It is an obstacle to the synthesis, decomposition, utilization and regulation of sugar in the body, which leads to abnormal increase or decrease of blood sugar level. It is the pathological and physiological basis of insulin resistance, hyperinsulinemia, diabetes and other diseases, and the two are mutually causal.

The mechanism of GMD is complex and diverse, mainly due to three reasons. One is excessive glucose intake, which prevents the body from utilizing it in a timely manner (Lascar et al., 2018). The second is the utilization of insulin. Insufficient insulin secretion leads to an increase in blood sugar levels (Kahn et al., 2006; Butler et al., 2013; Kahn et al., 2014). Furthermore, insulin resistance (IR) due to various factors such as genetic factors, obesity, lack of exercise, and inflammation also leads to an increase in blood sugar levels (Kahn et al., 2006; Petersen and Shulman, 2018). The third is that lipid metabolism disorders and adipocyte inflammation may also interfere with the body’s processing and utilization of glucose (Hotamisligil, 2017). Clinical manifestations of GMD include diabetes, impaired glucose tolerance (IGT), impaired fasting glucose (IFG), etc. Fasting plasma glucose (FPG), oral glucose tolerance test (OGTT), hemoglobin A1c (HbA1c) and other indicators are commonly used to diagnose glucose metabolism disorders (Davidson and Schriger, 2010). In addition, indicators such as homeostasis model assessment insulin resistance (HOMA-IR) and insulin levels can evaluate the status of insulin sensitivity and insulin secretion function, which is helpful for further diagnosis and treatment of GMD (Wallace et al., 2004). Clinical intervention of GMD mainly includes insulin sensitizer metformin, glucagon like peptide-1 (GLP-1) receptor agonist exenatide, which promotes insulin synthesis and secretion, and sodium-dependent glucose cotransporter protein 2 (SGLT2) inhibitor, which inhibits the reabsorption of glucose in proximal renal tubules, and has the ability to delay the breakdown and absorption of glucose in the intestine α-glycosidase inhibitors such as acarbose (Lazzaroni et al., 2021). However, they have certain side effects, such as lactoacidosis, vitamin B12 deficiency, digestive system discomfort, hypoglycemia, and other adverse reactions, especially in patients with liver and kidney dysfunction (Salpeter et al., 2010; Zhang F. et al., 2020; Sakyi et al., 2021).

Liver is not only the main organ of glucose and lipid metabolism, but also the main place of IR (Petersen and Shulman, 2018; Yang et al., 2018). When glucose metabolism is disrupted and blood sugar concentration is too high, it will stimulate insulin secretion, leading to a rapid increase in glucose transporter-4 (GLUT4) on the cell membranes of the liver, muscles, and adipose tissue, thereby quickly absorbing glucose from the blood, synthesizing glycogen, or converting it into fat for storage (Chadt and Al-Hasani, 2020). However, long-term hyperglycemia level exceeds the metabolic capacity of the liver, leading to liver damage, increased oxidative stress and inflammatory reaction, which leads to the disorder of malondialdehyde (MDA) and superoxide dismutase (SOD) (Song et al., 2022). Clinical research also points out that oxidative stress caused by high-fat diet promotes the inactivation of GLUT4 carbonylation to produce insulin resistance (Boden et al., 2015). In addition, long-term high blood sugar levels will also induce the accumulation of advanced glycation end products (AGEs) in the body, and a large amount of evidence suggests that AGEs can cause tissue damage, ß Cell damage can inhibit insulin secretion, directly lead to the combination of long-term complications of diabetes (Zhao et al., 2009), and reduce peripheral insulin sensitivity, resulting in insulin resistance (Uribarri et al., 2011; Cai et al., 2012). AGEs further increase intracellular reactive oxygen species (ROS) levels by binding to receptors for advanced glycation end products (RAGE), exacerbating inflammation and tissue damage (Vlassara and Uribarri, 2014). Meanwhile, the enhancement of ROS in turn contributes to the formation of AGEs and the expression of RAGE, which exacerbates the damage mediated by AGEs (Kang and Yang, 2020). Therefore, hyperglycemia stimulates oxidative stress in the body, which leads to the decline of GLUT4’s ability to transport glucose. At the same time, the accumulation of AGEs leads to the intensification of oxidative stress. In the long run, the ability of the liver to regulate glucose and lipid metabolism gradually decreases, which then leads to insulin resistance and diabetes.

*Gynostemma pentaphyllum* (Thunb.) Makino belongs Cucurbitaceae family and is widely distributed in Asian countries and used as tea and function food (Xie et al., 2012; Xia et al., 2020). Gypenosides (Gyp), saponins of *Gynostemma pentaphyllum*, are the main active components. Gypenosides after heat-processing (HGyp) have been suggested to exert various stronger biological effects, such as, antioxidant, anti-obesity and anti-tumor activities (Xing et al., 2019; Liu et al., 2021; Zu et al., 2021). At present, the effects of *G. pentaphyllum* on protecting overweight, hyperglycemia and hyperlipidemia have been reported (Norberg et al., 2004; Liu et al., 2017; Xie et al., 2023), while the research on the AGE-RAGE signaling pathway is only an *in vitro* experiment (Zhang H. et al., 2018), and there are no *in vivo* studies reported. Therefore, this study investigated the potential protective and antioxidant effects of HGyp on high fat diet-induced GMD in a mouse model combined with the blood components of HGyp and network pharmacology.

## 2 Materials and methods

### 2.1 Chemicals and reagents

*Gynostemma pentaphyllum* was bought at Zhangzhou, Fujian, China and was professionally identified. The sample of reference (No. GP, 2016-01) has been placed in the Isolation and Structure Identification Laboratory in School of Pharmacy, Minzu University of China. Hematoxylin-eosin staining (H&E) were acquired from Wuhan Servicebio Biotechnology Co., Ltd. (Wuhan, China). LDL-C, HDL-C, TCHO, TG, SOD, MDA, GSP were provided by Nanjing Jiancheng Bio-Engineering Institute Co., Ltd. (Nanjing, China). Glycogen was purchased from Beijing Solarbio Science and Technology Co., Ltd. (Beijing, China). Insulin and AGEs were bought from Bioswamp Life Science Lab (Wuhan, China). Glo1 polyclonal antibody and RAGE, GLUT4 monoclonal antibody were obtained from Proteintech Group (Rosemont, United States). ß-actin was purchased from Beijing Lablead Biotech Co., Ltd. (Beijing, China).

### 2.2 Preparation of HGyp samples

HGyp samples were prepared by our laboratory (Xie et al., 2022; Xie et al., 2023). The leaves of *G. pentaphyllum* were steamed at 120°C under 0.24 MPa for 3 h. After heat processing, they were reflux extracted with 80% ethanol (1:8, w/v) for 2 h for three times and then concentrated by rotary evaporator under reduced pressure. Ethanol extract was loaded to HP20 microporous resin and eluted with water, 50% and 95% ethanol successively. The 95% eluate, rich in gypenosides, was concentrated under reduced pressure to obtain total saponin of heat-processed *G. pentaphyllum* (HGyp).

### 2.3 Animals and experimental design

Five-week-old male C57BL/6J mice were acquired from Beijing Weitong Lihua Laboratory Animal Technology Co., LTD (License No. SCXK (jing) 2022-0002) with acclimatization for 7 days. During the experimental period, mice were kept in standard cages on their own on a 12-h light/dark cycle at controlled temperature (23 ± 2°C), humidity (50% ± 10%). After acclimatization, mice were then used to carry out the experiments. All animal protocols were approved (ECMUC2019003AO, 9 March 2019) by the Biological and Medical Ethics Committee of Minzu University of China and all animal work was carried out in accordance with the relevant guidelines and regulations.

After the time of adaptation, the mice were split into six groups (*n* = 10) during the experiment: normal diet group (NFD), high-fat diet group (HFD), metformin positive group at 200 mg/kg (Met), HGyp at 200 mg/kg (H-HGyp), HGyp at 100 mg/kg (M-HGyp) and HGyp at 50 mg/kg (L-HGyp)-treated groups. NFD group mice were given standard diet (20% protein; Sibeifu (Beijing) Biotechnology Co., LTD), while other mice were given high-fat diet (60% fat; Sibeifu (Beijing) Biotechnology Co., LTD) for 12 weeks. The mice were anesthetized and euthanized by intraperitoneal injection of 50 mg/kg pentobarbitone sodium. The blood samples were collected by orbital blood sampling. The blood, livers and kidneys were kept in −80°C for subsequent analysis.

### 2.4 Blood biochemical analysis

The blood sample was centrifuged at 3500 rpm for 15 min to obtain serum sample. HDL-C, LDL-C, TCHO, TG, GSP and insulin were measured using associated kits according to the manufacturer’s recommendations. The content of blood glucose was measured using Yuwell 550 glucometer (Yuwell, Danyang, China) by dropping blood to a glucose test strip.

Oral glucose tolerance test (OGTT) was performed by oral administration of 2.0 g/kg D-glucose to mice after fasting for 12 h. The blood samples were collected from the tip of the tail at 0, 30, 60, 90 and 120 min and measured the blood glucose levels.

### 2.5 Liver tissue biochemistry assays

The proper amount of liver was taken and the levels of glycogen, MDA, SOD and AGEs were measured through enzyme linked immunosorbent assay (ELISA) from the related kits according to the manufacturer’s recommendations.

### 2.6 Histological analysis

The sample was fixed in 4% paraformaldehyde solution for 48 h, then it was cut into 4 μm sections, embedded in paraffin, and refixed. Hematoxylin-eosin staining (H&E) begins with the removal of paraffin from xylene and dehydration, followed by 4 min of hematoxylin staining and 2 min of eosin staining. An optical microscope was used to view and take pictures of the sectioned tissues (DMIL LED; Leica, Wetzlar, Germany).

### 2.7 Identification of serum migrant compounds of HGyp

#### 2.7.1 Preparation of samples

The freeze-dried powder of HGyp was dissolved with methanol, centrifuged at 12,000 rpm for 15 min, and the supernatant was filtered through a 0.22 μm nylon filter membrane. Ten microliter of the filtrate was injected into the LC-MS for analysis.

HGyp was oral administrated to mice for 12 h and the blood was taken and stood for 2 h at 4°C. The blood was centrifugated at 3500 rpm for 15 min at 4°C. An equal amount of 100 μL supernatant was added to three times volumes of acetonitrile and vortexed for 5 min, the mixture was centrifugated at 12,000 rpm for 15 min at 4°C. The supernatant was added to 100 μL methanol and vortexed for 2 min, and centrifugated at 12,000 rpm for 15 min at 4°C. The supernatant was dried under nitrogen gas. The residue was dissolved in methanol and filtered through a 0.22 μm microporous membrane. Ten microliter of the filtrate was injected into the LC-MS for analysis.

#### 2.7.2 UPLC-MS/MS analysis

UPLC/MS analysis was performed using an ACQUITY UPLC System (Waters, Nebraska, United States) equipped with a Q/TOF-MS system (TOF™ 6600, AB SCIEX, Foster City, United States). HGyp and serum samples were separated by a Waters Acquity UPLC BEH 18 column (2.1 × 50 mm, 1.7 μm) kept at 30°C. The mobile phases consisted of 0.1% formic acid aqueous solution (A) and acetonitrile (B). The gradient elution condition was as follows: 0−7 min, 20%–25% B; 7−35 min, 25%–70% B; 35−40 min, 70%–95%. The flow rate was 0.3 mL/min. Mass analysis was carried out in negative mode with −4.5 kV of ion source interface voltage and 500°C of the ion source temperature. Nitrogen was used as both the drying gas and the atomizing gas. The scan range was *m/z* 100–1,200 and the fragment voltage was set at 60 V.

### 2.8 Plasma metabolomics analysis

#### 2.8.1 Preparation of samples

Mice were fasted in advance for 12 h, and the blood was taken and stood for 2 h at 4°C. The blood was centrifugated at 3,500 rpm for 15 min at 4°C. An equal amount of 100 μL supernatant was added to 3 times volumes of 80% methanol and vortexed for 5 min, the mixture was stood for 30 min at −20°C and centrifugated at 14,000 *g* for 10 min. The supernatant was filtered through a 0.46 μm nylon filter membrane. Twenty microliter of the filtrate was injected into the LC-MS for analysis. An equal amount of supernatant was taken from all the samples and mixed into QC samples for detection.

#### 2.8.2 UPLC-MS/MS analysis

UPLC/MS analysis was performed using a Thermo Scientific™ Dionex™ ΜltiMate™ 3000 Rapid Separation LC (Thermo, Massachusetts, United States) equipped with a Q Exactive system (Thermo, Massachusetts, United States). Plasma samples were separated by a Waters Acquity UPLC BEH C8 column (2.1 × 100 mm, 1.7 μm) kept at 30°C. The mobile phase A was acetonitrile/water (60/40), and the mobile phase B was isopropanol/acetonitrile (90/10). Both A and B contained 0.1% formic acid and 10 mmol/L ammonium formate. The gradient elution condition was as follows: 0−1 min, 98% B; 1−5 min, 98%–30% B; 5−8 min, 30%–0% B; 8−14 min, 0% B. The flow rate was 0.25 mL/min. Mass analysis was carried out in both positive and negative mode with 2.5 kV of spray voltage and 320°C of the capillary temperature. The scan range was *m/z* 100-1500.

### 2.9 Network analysis

Network analysis was used to predict the targets and pathways of the serum migrant compounds of HGyp against GMD. Genecards (https://www.genecards.org/) and the National Center for Biotechnology Information (NCBI) (https://www.ncbi.nlm.nih.gov/) were used to search for the genes associated with the search phrases “glucose metabolic diseases.” Only research on *homo sapiens* are included in the search results, possible protein targets for serum migrant drugs were obtained from swisstarget (http://www.swisstargetprediction.ch/). Using the venny program (https://bioinfogp.cnb.csic.es/tools/venny/) to upload the protein of serum migratory chemicals possible targets and genes connected to glucose metabolic diseases. Using string to build networks and pathways based on how genes and proteins interact. For illness target Gene Ontology (GO) enrichment and network analysis, R (version 4.0.4 for Windows) and Cytoscape 3.7.0 (http://www.cytoscape.org) were used.

### 2.10 Western blotting analysis

RIPA lysis buffer (Servicebio, Wuhan, China) was used to homogenize and extract the liver tissues. Lysates were separated by centrifugation at 12,000 rpm for 10 min at 4°C. BCA reagent was used to quantify the supernatant using the same volumes of lysates (Lablead, Beijing, China). Polyvinylidene difluoride (PVDF) membranes were used to transfer the separated proteins after SDS-PAGE separation. GLO1, RAGE, GLUT4, and ß-actin were detected using primary antibodies on the membranes. The membranes were treated with secondary antibodies containing horseradish peroxidase (HRP) for 1 h after being rinsed with Tris-buffered saline and 0.1% Tween 20 (TBST). TBST was used to clean the membranes, an enhanced chemiluminescence (ECL) solution (Lablead, Beijing, China), was used to treat them, and a Tanon 4200SF western scanner was used to detect them (Tanon, Shanghai, China). The target bands’ densities were measured using ImageJ software.

### 2.11 Statistical analysis

A statistical tool was used to calculate each experiment’s mean and standard deviations (mean ± SD). Difference between groups were analyzed by *t*-test and one-way analysis of variance (ANOVA). Statistical significance was defined as *p* < 0.05. The statistical analysis was performed using GraphPad Prism 8 (GraphPad Software, San Diego, CA, United States). PeakView 1.2 software was carried out the LC-MS analysis.

## 3 Results

### 3.1 Effects of HGyp on HFD-induced GMD symptoms

#### 3.1.1 Effects of HGyp on physiological changes

**FIGURE 1:**
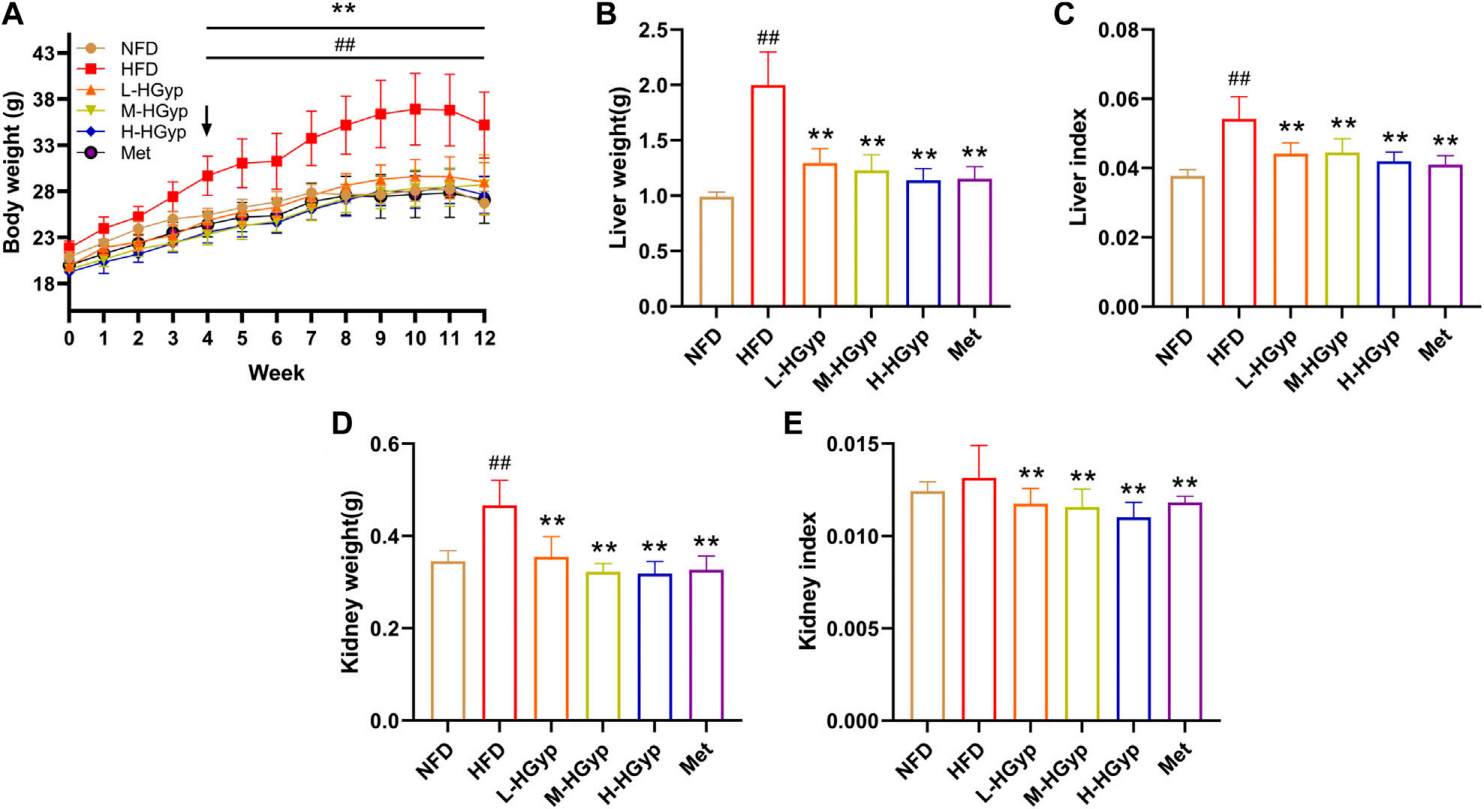
Effects of HGyp on serum of HFD-induced GMD mice. NFD: normal control group; HFD: high fat diet group; L-HGyp: HFD with 50 mg/kg HGyp group; M-HGyp: HFD with 100 mg/kg HGyp group; H-HGyp: HFD with 200 mg/kg HGyp group; Met: HFD with metformin group. **(A)** Body weight, **(B)** Liver weight, **(C)** Liver index, **(D)** Kidney weight, **(E)** Kidney index. Data are presented as mean ± SD (*n* = 10). ^##^ *p* < 0.01 vs. NFD group. **p < 0.01 vs. HFD group.

The body weight of mice increased gradually in the first 11 weeks, particularly in the HFD group. Body weight in the HFD group considerably increased compared to the NFD group (*p* < 0.01). Compared with the HFD group, L-HGyp, M-HGyp and H-HGyp decreased significantly (*p* < 0.01) in body weight (Figure 1A). After 12th week, the body weight of HFD was 35.2 ± 3.57 g, increased compared with NFD (26.71 ± 1.29 g) significantly (*p* < 0.01). Compared with HFD, the body weights of L-HGyp, M-HGyp and H-HGyp decreased to 28.99 ± 2.13, 28.74 ± 3.21 and 27.62 ± 1.99 g (*p* < 0.01), respectively.

In HFD group, the liver weight, liver index and kidney weight were higher than that in NFD group significantly (*p* < 0.01). The liver weight, liver index and kidney weight of L-HGyp, M-HGyp and H-HGyp groups were considerably lower than those of the HFD group (*p* < 0.01) (Figures 1B–D). Additionally, L-HGyp, M-HGyp and H-HGyp groups significantly underperformed the kidney index compared to the HFD group (*p* < 0.01), whereas the kidney index in the HFD group was higher than that of the NFD group (Figure 1E).

#### 3.1.2 Effects of HGyp on blood lipids changes

Several serum parameters including TCHO, TG, HDL-C and LDL-C were measured in order to evaluate the impact of HGyp on serological alterations in GMD mice (Table 1). The levels of TCHO (*p* < 0.01), TG (*p* < 0.01), HDL-C (*p* < 0.05), and LDL-C (*p* < 0.01) in the HFD group were noticeably higher than those in the NFD group. Comparative to the HFD group, H-HGyp reduced the levels of TCHO, TG and LDL-C considerably (*p* < 0.01). In comparison to the HFD group, the levels of TG (*p* < 0.01) and LDL-C (*p* < 0.01) were significantly lower in HGyp groups.

**TABLE 1:** Effects of HGyp on lipid levels of HFD-induced GMD in mice (mean ± SD, *n* = 10).

Group	NFD	HFD	L-HGyp	M-HGyp	H-HGyp	Met
TCHO (mmol/L)	2.88 ± 0.25	5.08 ± 0.49^##^	4.69 ± 0.48	4.50 ± 0.29	3.98 ± 0.29**	4.13 ± 0.20**
TG (mmol/L)	1.26 ± 0.06	2.04 ± 0.08^##^	1.73 ± 0.07**	1.49 ± 0.06**	1.36 ± 0.05**	1.39 ± 0.05**
HDL-C (mmol/L)	4.87 ± 0.62	6.64 ± 0.85^#^	6.53 ± 0.80	7.50 ± 0.57	7.55 ± 0.47	7.41 ± 0.84
LDL-C (mmol/L)	0.48 ± 0.07	1.50 ± 0.09^##^	1.04 ± 0.08**	0.89 ± 0.04**	0.70 ± 0.07**	0.73 ± 0.09**

^#^ *p* < 0.05. ^##^ *p* < 0.01 vs. NFD, group. **p < 0.01 vs. HFD, group.

#### 3.1.3 Effects of HGyp on glucose metabolism changes

**FIGURE 2:**
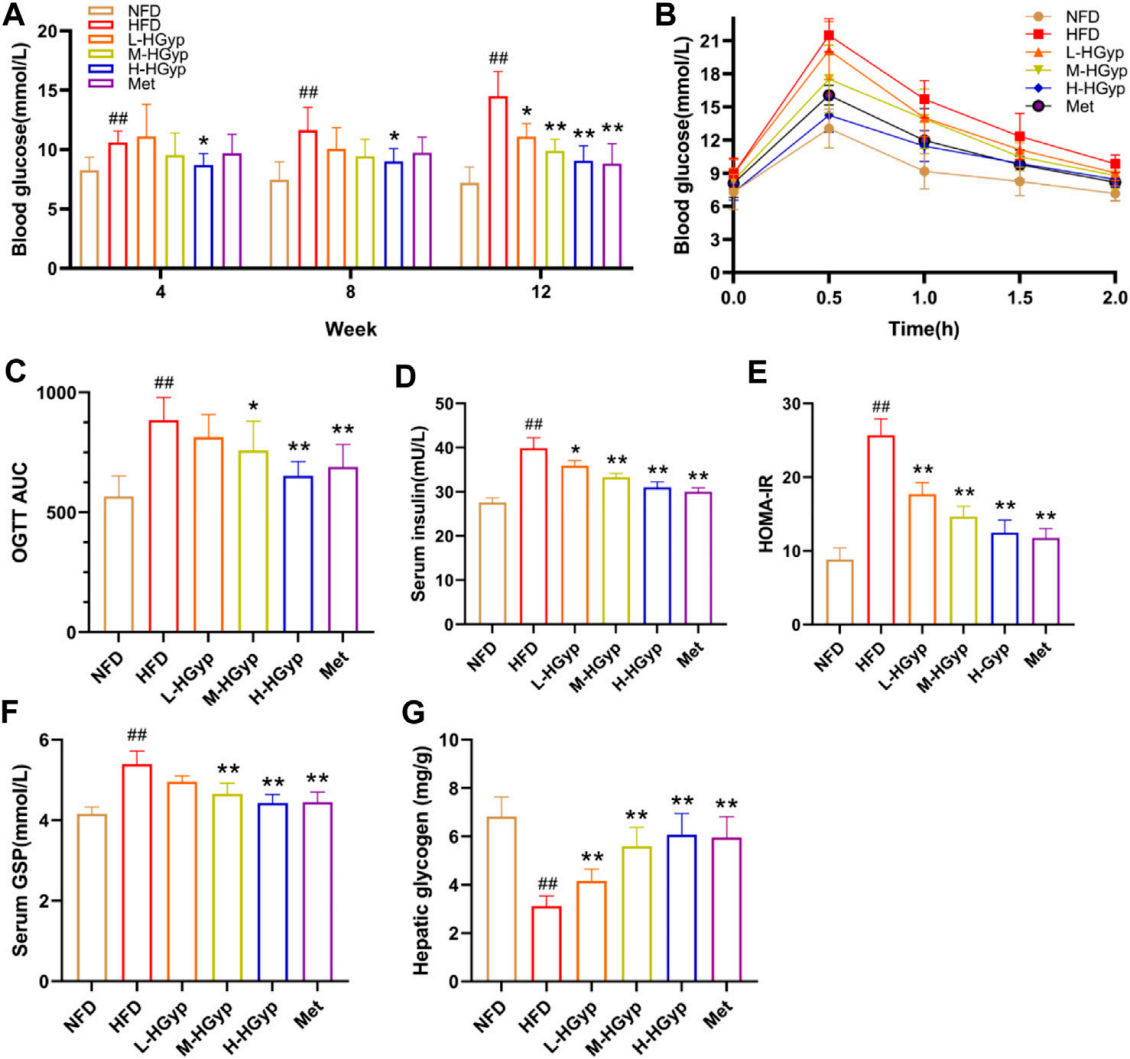
Effects of HGyp on HFD-induced GMD mice. **(A)** Contents of blood glucose, **(B)** AUC, **(C)** OGTT AUC, **(D)** contents of insulin, **(E)** HOMA-IR, **(F)** contents of GSP, **(G)** content of hepatic glycogen. Data are presented as mean ± SD (*n* = 10). ^##^ *p* < 0.01 vs. NFD group. *p < 0.05, **p < 0.01 vs. HFD group.

Over the course of 12 weeks, the glucose levels in GMD mice gradually climbed. In the final test, L-HGyp (*p* < 0.05), M-HGyp (*p* < 0.01), and H-HGyp (*p* < 0.01) groups significantly decreased compared to the HFD group (Figure 2A). The impact of HGyp on glucose metabolism was then assessed using OGTT tests. The OGTT showed that mice treated with the HGyp exhibited dose-dependently improved glucose tolerance (Figures 2B, C). The results of HOMA-IR were greatly decreased as a result of HGyp since it also significantly increased insulin levels (*p* < 0.01; Figures 2D, E). Levels of GSP (*p* < 0.01; Figure 2F) and hepatic glycogen (*p* < 0.01; Figure 2G) were considerably higher in the M-HGyp and H-HGyp groups compared to the HFD group.

### 3.2 Effects of HGyp on HFD-induced liver damage

**FIGURE 3:**
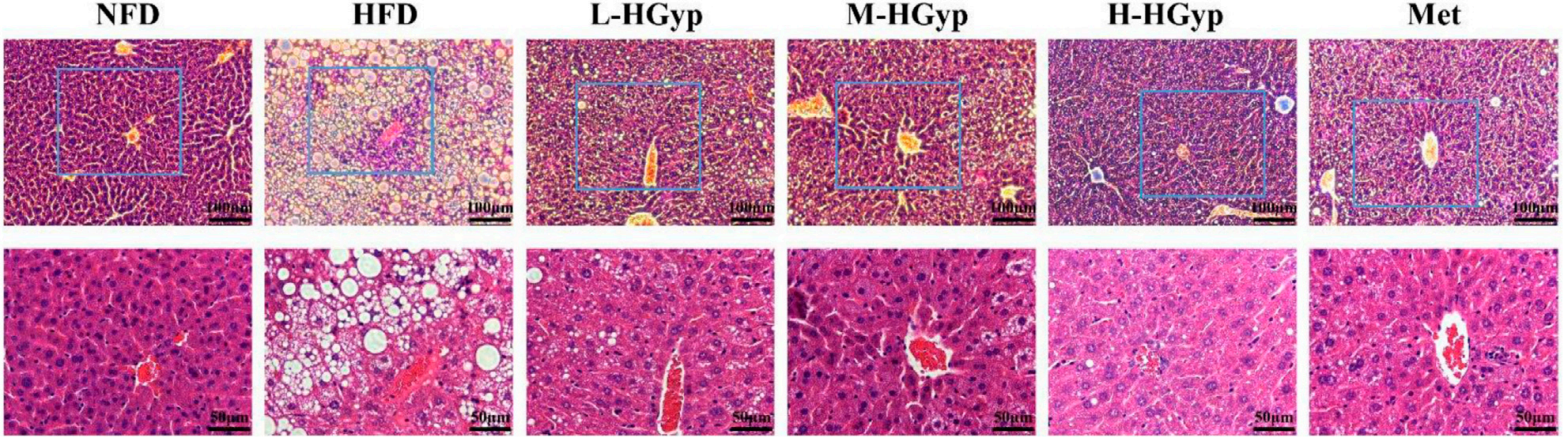
Representative histological findings of liver sections after H&E staining.

The structural damage to the liver tissue was shown using H&E staining. Hepatocytes from mice in the NFD group are nicely distributed, as seen in Figure 3. Hepatocytes along the central vein in the HFD group had significant steatosis, bullous steatosis, vacuolar degeneration, and congestion of the hepatic sinuses. There was also a tiny localized infiltration of inflammatory cells surrounding the vein. Lipid droplets of various sizes are seen inside hepatocytes. When L-HGyp, M-HGyp, and H-HGyp were administered to the HFD group, these liver histological abnormalities were reduced, and the H-HGyp group in particular almost recovered to the NFD group.

### 3.3 Effects of HGyp on antioxidant factors in HFD-induced GMD mice

**FIGURE 4:**
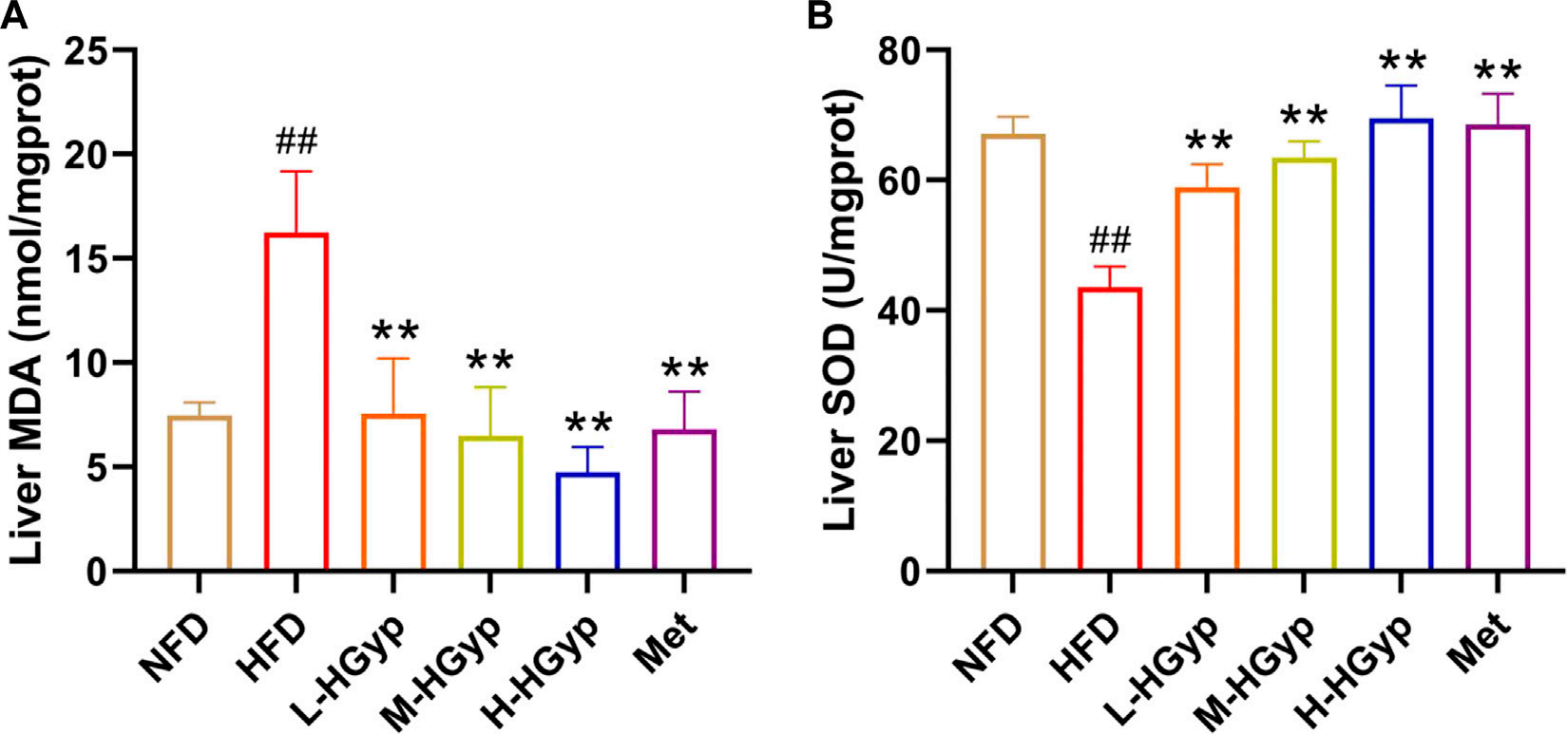
Effects of HGyp on antioxidant factors of HFD-induced GMD in mice. **(A)** Contents of MDA, **(B)** contents of SOD. Data are presented as mean ± SD (*n* = 10). ^##^ *p* < 0.01 vs. NFD group. **p < 0.01 vs. HFD group.

Oxidative damage to the liver can lead to an increase in MDA levels and a decrease in SOD levels. The MDA and SOD in the liver were measured through ELISA assay in order detect the antioxidant effects of HGyp on GMD mice. Compared to NFD group, the level of MDA of liver was significantly increased in the HFD group, whereas L-HGyp, M-HGyp, and H-HGyp groups decreased the MDA levels compared to HFD group (*p* < 0.01) (Figure 4A). The level of SOD of liver in HFD group was decreased significantly compared to NFD group, whereas L-HGyp, M-HGyp, and H-HGyp groups increased the levels of SOD significantly compared to HFD group (*p* < 0.01) (Figure 4B).

### 3.4 Analysis of serum migrant compounds after administration of HGyp

**FIGURE 5:**
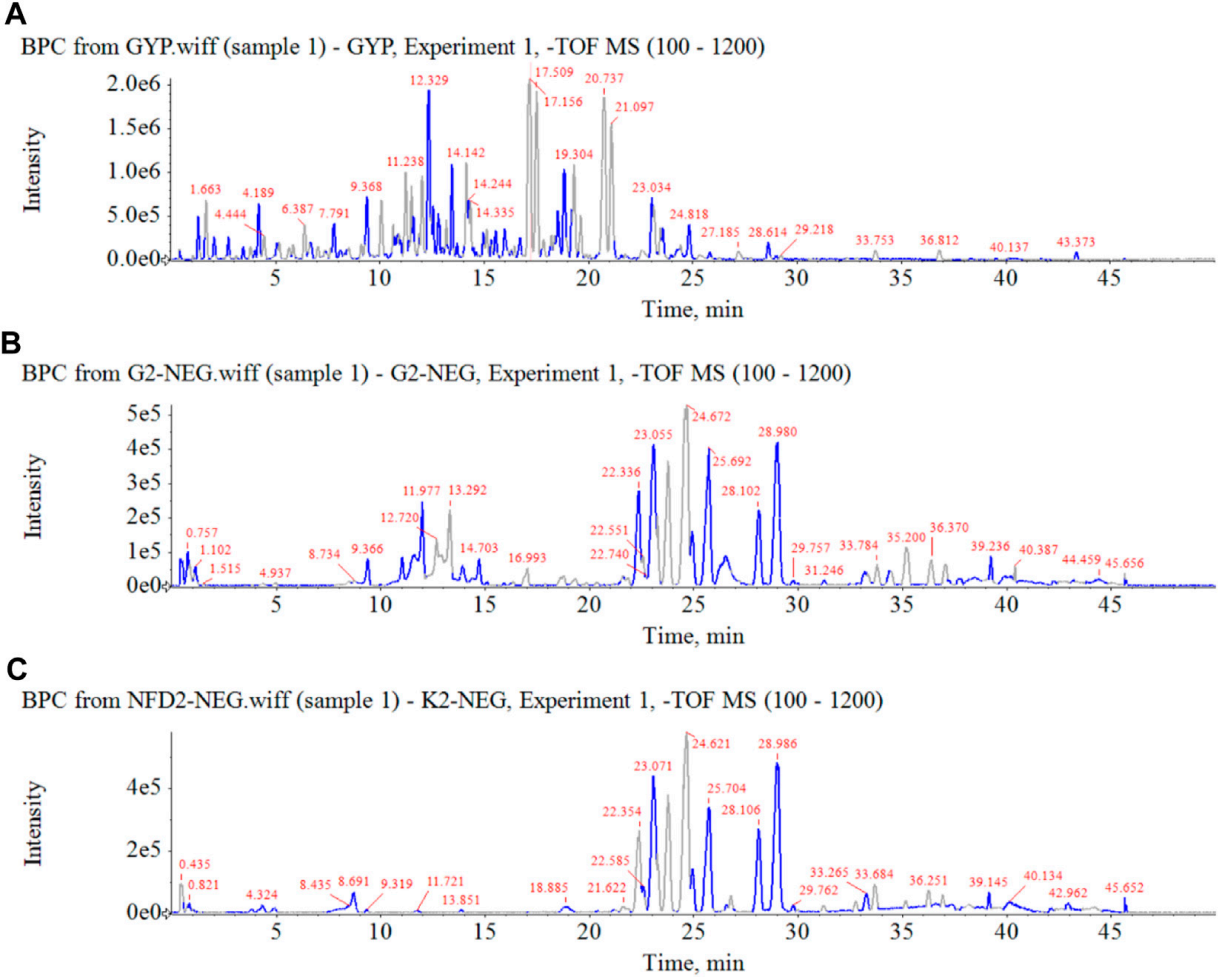
Base peak chromatograms of HGyp in negative ion mode. **(A)** HGyp, **(B)** serum sample collected from normal C57BL/6J mice after oral administration of HGyp, **(C)** serum sample (control serum) collected from normal C57BL/6J mice.

**FIGURE 6:**
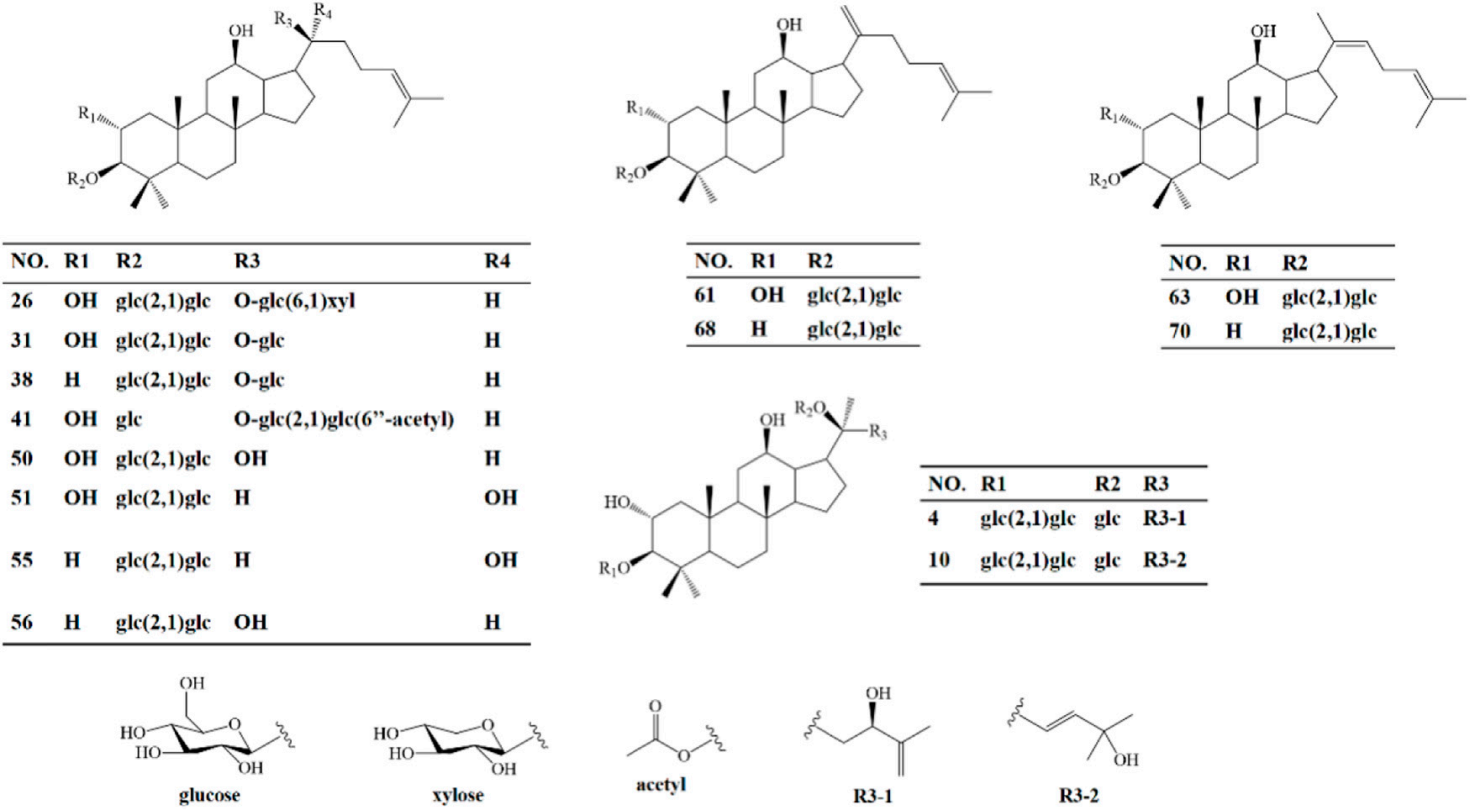
Structure of 14 serum migrant compounds.

In order to clarify the serum migrant compounds after administration of HGyp to mice, LC-MS was used for analysis in negative mode. The results showed that there were 73 gypenosides identified from the base peak chromatogram (BPC) (Figure 5A) using standards and associated literature (Supplementary Table S1). Twenty-four gypenosides were detected from serum after administration of HGyp to mice (Figure 5B) compared to control serum (Figure 5C). From them, 14 major gypenosids (Figure 6) with known structures were selected for network analysis (Supplementary Table S2).

### 3.5 Metabolomic analysis of HFD-induced GMD in mice treated with HGyp

**FIGURE 7:**
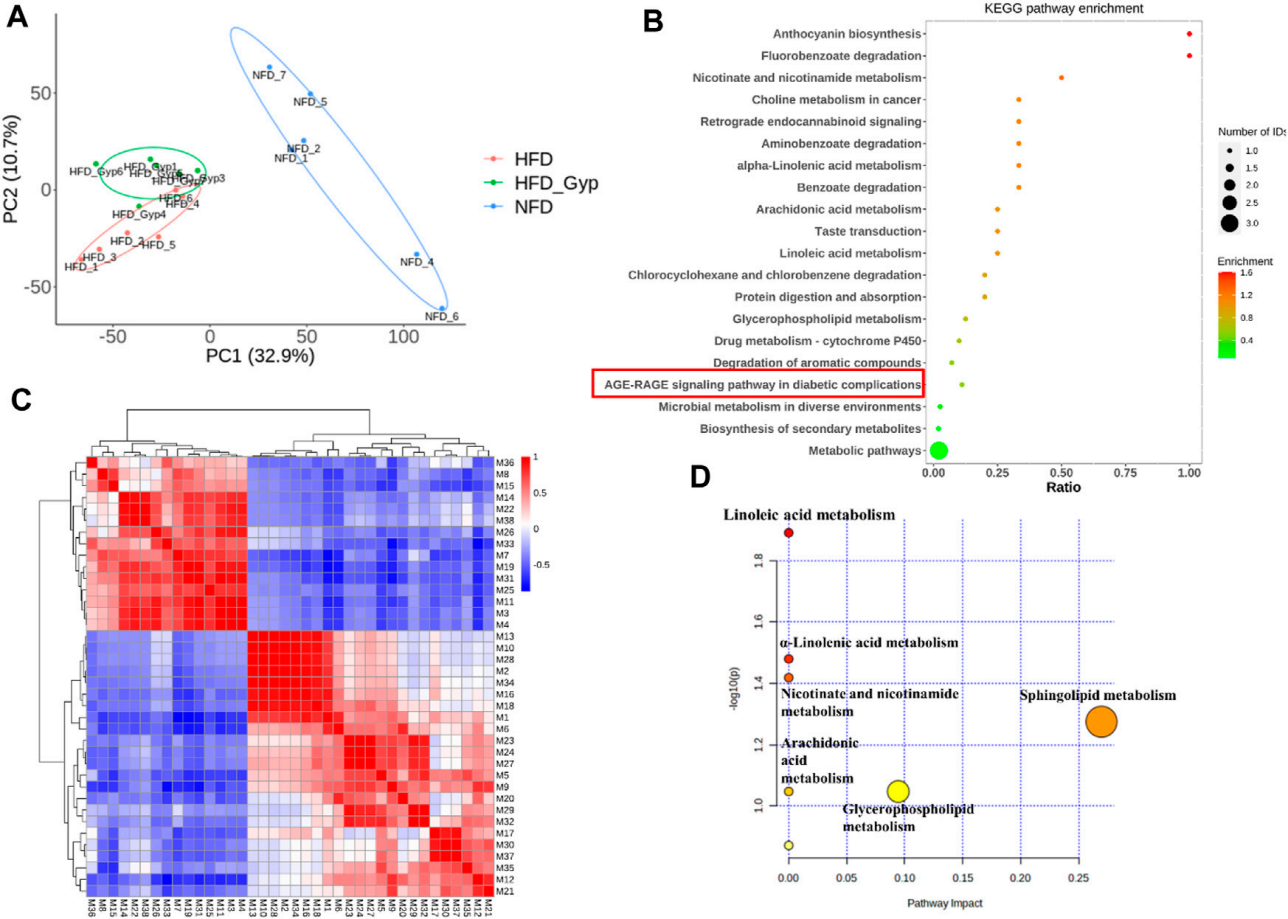
Metabolomic analysis of HFD-induced GMD in mice treated with HGyp in positive ion model. **(A)** PCA analysis. Blue, red and green represented NFD, HFD and H-HGyp group, respectively (*n* = 6). **(B)** KEGG pathway enrichment analysis. The horizontal axis represents the ratio, the vertical axis represents each GO entry. The color represents the enrichment (- log10 (*p*-value)), and the size of the circle represents the metabolic number. **(C)** Pearson correlation analysis. Red and blue represented positive and negative correlation, respectively. The darker the color, the stronger the correlation between two biomarkers. **(D)** Metabolic pathway analysis.

The total ion chromatograms were obtained from QC, control, model and HGyp group using UPLC-Q-TOF/MS. A total 9,110 and 8,600 ions were extracted from the positive and negative datasets, respectively. Principal component analysis (PCA) of 3 groups in positive ion model showed that HGyp treatment had effect on the metabolic profile of HFD-induced GMD in mice (Figure 7A). After normalization of quantitative values, the fold change (FC) is the ratio of the mean of repeated quantitative values of all organisms in the comparative group for each metabolite, and the *p*-value of *t*-test is used to search for differentially expressed metabolites. Set the threshold to VIP >1.0, difference multiple FC > 2.0 or FC < 0.5 and *p*-value <0.05 to screen out differential metabolites. A total of 38 potential metabolites in HGyp group could be recalled to the content compared with model group in positive ion model (Table 2).

**TABLE 2:** Identified differential metabolites in serum between HFD group and H-HGyp group in positive ion model.

NO.	HMDB ID	Melecular formula	*m/z*	Rt/min	VIP	*p*-value	Vs. HFD
M1	HMDB0041410	C_12_H_14_O_3_	413.1967	8.18	1.42	<0.001	Up
M2	HMDB0039642	C_30_H_48_O_2_	441.3716	8.00	2.88	<0.001	Up
M3	HMDB39785	C_28_H_52_O_8_	1033.7398	6.01	1.52	<0.001	Down
M4	HMDB0038029	C_25_H_40_O_11_	1033.5173	6.02	1.60	<0.001	Down
M5	HMDB0014727	C_20_H_26_N_4_O	361.1996	6.16	2.55	<0.001	Up
M6	HMDB0004193	C_7_H_8_N_2_O_2_	153.0655	3.53	1.14	<0.001	Up
M7	HMDB0030402	C_5_H_9_N_3_O_3_	319.1356	4.32	1.65	<0.001	Down
M8	HMDB0035671	C_21_H_42_O_2_	327.3249	7.92	3.26	<0.001	Down
M9	HMDB0004978	C_48_H_93_NO_8_	834.6765	14.76	1.24	0.0012	Up
M10	HMDB0039700	C_28_H_48_O	423.3605	8.02	1.25	0.0016	Up
M11	HMDB0033679	C_28_H_33_O_14_^+^	616.1745	6.29	1.66	0.0020	Down
M12	HMDB0029495	C_12_H_12_O_2_	189.0905	6.11	1.29	0.0023	Up
M13	HMDB0038244	C_30_H_52_O_4_	477.3924	7.60	12.47	0.0024	Up
M14	HMDB0060654	C_20_H_21_FN_2_O_2_	363.1475	4.77	2.94	0.0033	Down
M15	HMDB0039048	C_23_H_22_O_7_	821.2766	5.74	1.68	0.0038	Down
M16	HMDB0041135	C_28_H_48_O_5_	487.3372	7.07	1.53	0.0053	Up
M17	HMDB0041051	C_44_H_72_O_18_	911.4615	5.09	1.60	0.0070	Up
M18	HMDB0060137	C_27_H_46_O_5_	473.3247	6.59	8.83	0.0087	Up
M19	HMDB0041766	C_28_H_33_O_16_^+^	648.1654	6.18	7.06	0.0169	Down
M20	HMDB0059780	C_13_H_17_N_5_O_4_	308.1347	4.26	1.31	0.0174	Up
M21	HMDB0041401	C_21_H_24_O_5_	713.3302	4.97	1.46	0.0178	Up
M22	HMDB0034688	C_19_H_14_O_3_	313.0844	5.26	1.45	0.0199	Down
M23	HMDB0008758	C_48_H_96_NO_8_P	868.6757	14.91	2.00	0.0203	Up
M24	HMDB0008795	C_50_H_96_NO_8_P	892.6741	14.73	1.60	0.0210	Up
M25	HMDB0060348	C_6_H_6_O_5_	159.0285	2.07	2.06	0.0212	Down
M26	HMDB0004949	C_34_H_67_NO_3_	1076.0263	6.08	1.03	0.0224	Down
M27	HMDB0011380	C_43_H_86_NO_7_P	782.6016	14.02	1.01	0.0239	Up
M28	HMDB0041533	C_30_H_50_O_3_	459.3820	8.01	6.31	0.0248	Up
M29	HMDB0029225	C_18_H_27_N_3_O_4_	699.4060	5.74	2.93	0.0255	Up
M30	HMDB0033237	C_16_H_30_O_10_	765.3758	5.11	1.54	0.0275	Up
M31	HMDB0041753	C_28_H_33_O_15_^+^	632.1698	6.17	8.87	0.0283	Down
M32	HMDB0002492	C_24_H_40_O_3_	399.2880	7.05	1.22	0.0289	Up
M33	HMDB0041970	C_19_H_21_N_5_O_3_S	422.1271	5.77	8.08	0.0306	Down
M34	HMDB0004947	C_30_H_59_NO_3_	504.4401	8.33	5.50	0.0384	Up
M35	HMDB0040700	C_17_H_23_NO_3_	290.1743	6.59	7.77	0.0407	Up
M36	HMDB0031721	C_14_H_20_O_9_	333.1186	3.98	7.34	0.0409	Down
M37	HMDB0034660	C_30_H_32_O_7_	505.2242	3.99	1.63	0.0423	Up
M38	HMDB0014850	C_15_H_13_FO_2_	267.0791	5.95	1.41	0.0460	Down

In addition, the identified metabolites and differential metabolites data of H-HGyp and HFD group of samples were uploaded to the website KEGG (http:www.genome.jp/kegg). The enrichment results of all identified proteins in the KEGG pathway showed that HGyp involved AGE-RAGE signaling pathway in diabetic complications (Figure 7B). Pearson correlation analysis showed that some metabolites were positive or negative correlated, such as the positive correlation between M13 and M10 was strong, and the negative correlation between M1 and M7 was strong. Therefore, it was suggested that these differential metabolites are interrelated (Figure 7C). The 38 differential metabolites between the H-HGyp group and HFD group were imported into the Metaboanalyst 5.0 (https://www.metaboanalyst.ca/faces/home.xhtml) to explore metabolic pathway analysis. The results showed that sphingolipid metabolism and glycerophospholipid metabolism were the crucial pathway for the HGyp on the HFD-induced GMD mice (Figure 7D).

### 3.6 Pathway analysis

**FIGURE 8:**
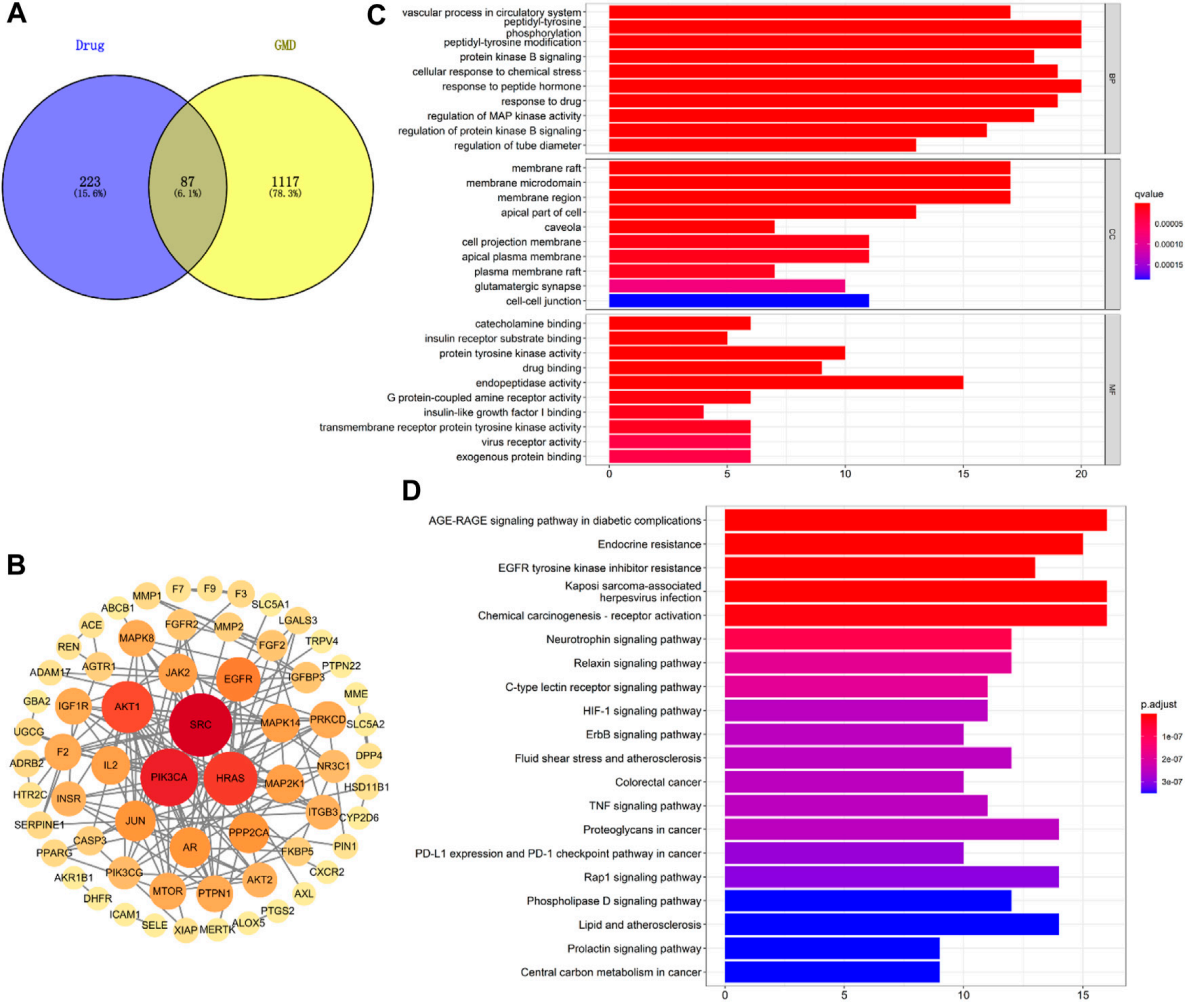
Network analysis of HGyp on GMD. **(A)** Wayne diagram of the intersection of gypenosides-related gene and GMD-related gene; **(B)** PPI network of 87 targets; **(C)** GO enrichment analysis of the identified targets in terms of biological process, cellular component and molecular function; **(D)** KEGG pathways enrichment results in relation to the potential targets of gypenosides.

The possible pathways of HGyp on HFD-induced GMD in mice were predicted using 14 serum migrant gypenosides and GMD related genes. The GeneCards Database and NCBI database yielded a total of 1,204 genes associated with GMD. Swiss Target database gathered the 310 predicted genes of the serum migrant gypenoside chemicals. The intersection of ingredient-related genes and GMD-related targets represented the 87 potential active targets of gypenosides on GMD (Figure 8A). The link between the various target genes was investigated using a PPI network that was built using the 87 targets that were loaded into the STRING database. Gypenosides inhibitory GMD targets included 64 genes through 161 interactions in the PPI network (Figure 8B). SRC (22), PIK3CA (19), HRAS (17), AKT1 (16), EGFR (12), and others have relatively greater degrees than others, according to PPI network (Supplementary Table S3).

Based on the *q* value with rich functionalities, the first 10 biological functions from the collected intersection genes were chosen for investigation using GO analysis (Figure 8C). The findings demonstrated that BP enrichment had a major role in peptidyl-tyrosine phosphorylation, peptidyl-tyrosine modification, and response to peptide hormone. The membrane raft, membrane microdomain and region were the primary sites of CC enrichment. And there was a direct correlation between MF enrichment and endopeptidase activity (Supplementary Table S4). There were 100 enriched pathways when KEGG pathway annotation clusters were examined. Listed below are the top 20 paths (Figure 8D). The top 20 pathways were chosen for further study based on *p*-value after the enrichment analysis for genes was conducted. The findings of KEGG pathway enrichment analysis supported the involvement of the AGE-RAGE signaling pathway in diabetes complications (Supplementary Table S5).

### 3.7 Effects of Hgyp on AGE-RAGE signaling pathway and GLUT4 monoclonal antibody in HFD-induced GMD mice

**FIGURE 9:**
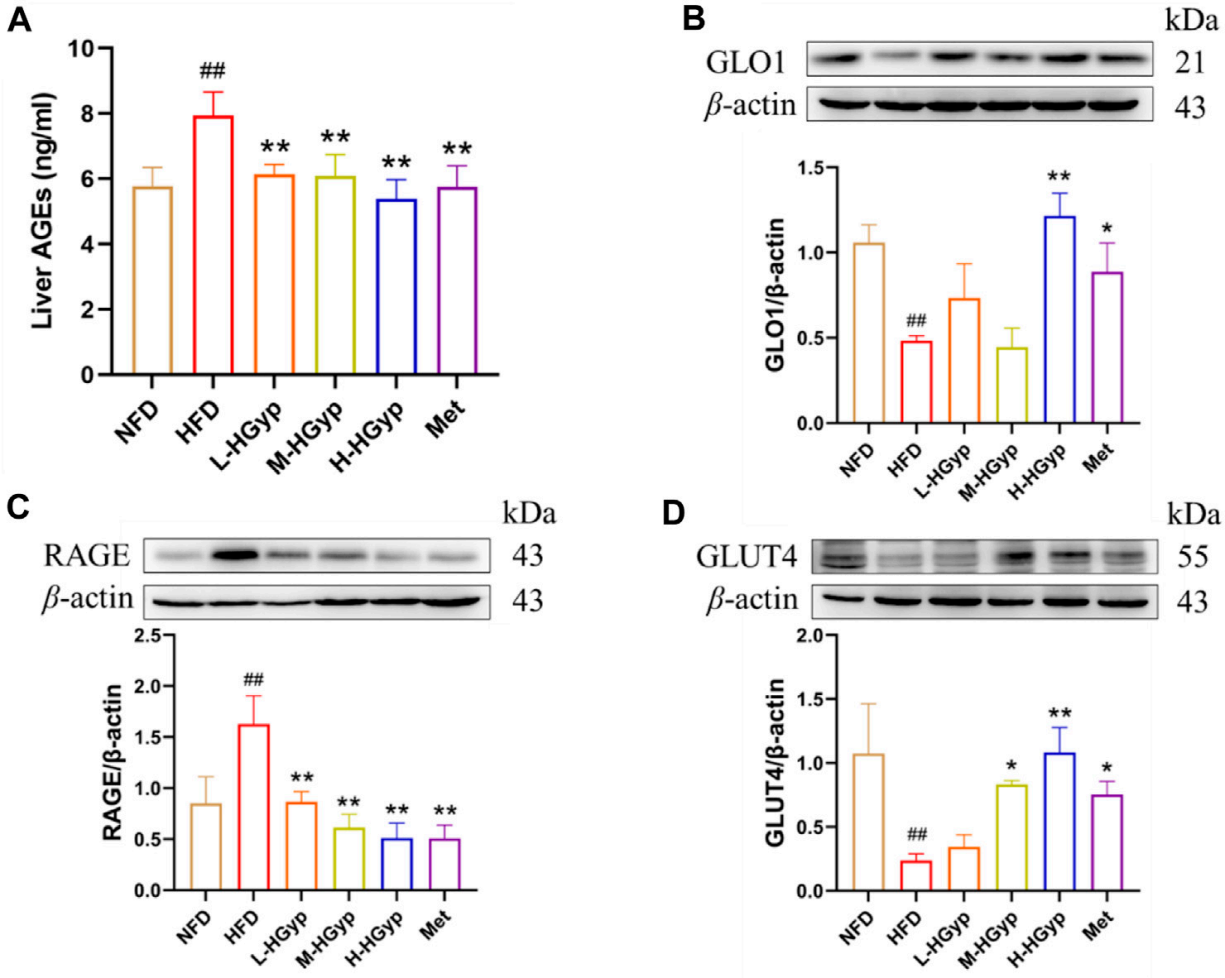
Effects of HGyp on AGE-RAGE signaling pathway and GLUT4 monoclonal antibody in HFD-induced GMD mice. **(A)** Contents of AGEs, Data are presented as mean ± SD (*n* = 10). **(B)** GLO1, **(C)** RAGE, **(D)** GLUT4, data are presented as mean ± SD (*n* = 3). ^##^ *p* < 0.01 vs. NFD group. *p < 0.05, **p < 0.01 vs. HFD group.

The content of AGEs in mouse serum was evaluated by the Elisa kit, and the levels of GLO1, RAGE, and GLUT4 in the liver were identified by Western blot, in order to confirm that HGyp intervenes in HFD-induced GMD mice through the AGE-RAGE signaling pathway. According to the findings, the content of AGEs was considerably higher in the HFD group (*p* < 0.01) and significantly lower in all treatment groups (*p* < 0.01) (Figure 9A). As compared to the NFD group, the HFD group levels of GLO1 and GLUT4 was lower but its level of RAGE was higher (*p* < 0.01) (Figures 9B–D). In comparison to the HFD group, the expression of GLO1 and GLUT4 in the H-HGyp group was up (*p* < 0.01), while the expression of RAGE in the HGyp groups was decreased (*p* < 0.01) (Figures 9B–D).

## 4 Discussion

Heat-processing can increase chemical diversity and biological activities of materials. For example, ginsenoside Rg3, Rg5 and Rk1, the active components from *Panax ginseng*, were produced more by heat-processing, and they have stronger activities, such as, anti-inflammatory and anti-tumor activities (Lee et al., 2012; Lee, 2014; Park et al., 2015; Shin et al., 2015). *Gynostemma pentaphyllum* (called Jiaogulan in Chinese) is distributed in China, Japan, Korea, and southeast Asian countries (Wang et al., 2017). It has been widely used as an edible and medicinal plant for thousands of years since the Ming dynasty in China. The leaves of *G. pentaphyllum* are often used in tea and contain a variety of biological functional components, which can be classified as saponins, flavonoids, polysaccharides, etc (Kao et al., 2008; Kim and Han, 2011; Ren et al., 2019). Gypenosides (Gyp) are the main constituents of *G. pentaphyllum* and gypenosides of heat-processed *G. pentaphyllum* (HGyp) have been found to exhibit biological activities, including lipid-lowering, glucose lowering and anti-tumor activities (Li et al., 2016; Khan et al., 2019; Xia et al., 2020; Xie et al., 2022). Therefore, in this experiment, the blood composition of HGyp was studied by means of serum pharmacochemistry. The results showed that 24 components of HGyp were found, among which 14 were the main components (Figure 6).

Network analysis was used to study the interactions between drugs and proteins or genes and diseases. It can describe the complexity of biological systems, drugs and diseases from a network perspective (Jiashuo et al., 2022). Here, we integrated information from a publicly available database to predict interactions between 14 gypenosides entered the blood and their potential targets in GMD, as well as associated networks and signaling pathways. We identified 14 saponin of the blood components. Using bioinformatics methods, we found the following. First, pathway analysis showed that gypenosides regulated AGE-RAGE signaling pathway. Secondly, it can be seen from the GO analysis results that genes related to sugar metabolism are reflected in the three processes, especially those related to sugar receptors and insulin. Finally, 87 genes were screened from the protein network map. Some have been linked to sugar metabolism, others have not been reported in detail. We predicted that HGyp might play a role through insulin-related AGE-RAGE pathways as well as oxidation-related pathways and glucose transport processes. This study demonstrated the expression levels of AGE, RAGE, GLUT4 and other proteins. Only a limited number of predictive molecular mechanisms have been verified in this study, and other targets, effects or signaling pathways need to be further investigated.

T2DM is a chronic metabolic disorder syndrome and the fourth cause of death in the world. It is characterized by lipid metabolic disorders and GMD, which leads to IR and hyperglycemia. IR is caused by insulin target cells, such as hepatocytes, skeletal muscle cells and adipocytes, which respond to insulin stimulation (Yang et al., 2018; Rachdaoui, 2020; Saltiel, 2021). In addition, oxidative stress is one of the important factors leading to IR. The imbalance of free radicals and antioxidants lead to oxidative stress and the decrease of peripheral insulin sensitivity, induce the production of inflammatory factors, and then promote the occurrence and development of diabetes and its complications by regulating IR (Zhang P. et al., 2020). Superoxide dismutase (SOD) is a key cellular antioxidant in oxidative stress, and its level can indirectly reflect the ability of the body to scavenge free radicals. IR and oxidative stress play important roles in the pathological process of T2DM. Clinically, hyperglycemia, hyperlipidemia, high OGTT, hyperinsulinemia and high homeostasis model assessment of insulin resistance (HOMA-IR) are the main manifestations (Phillips, 2012; Tang et al., 2015). In the mouse IR model, the mice showed glucose metabolism disorder related phenotypes such as elevated blood glucose, serum insulin, GSP and abnormal glucose tolerance (Xu F. et al., 2022). The long-term abnormal blood glucose level accelerates the progress of diabetes and the occurrence of complications. The abnormal blood lipid and vascular remodeling caused by it are important risk factors for cardiovascular and cerebrovascular diseases (Yamagishi, 2019). Early intervention of IR in patients with type 2 diabetes is considered to be the most effective strategy for the treatment of T2DM (Pandey et al., 2015). The commonly used hypoglycemic drugs include: biguanides metformin (the first-line agent) (Flory and Lipska, 2019; Bharath and Nikolajczyk, 2021), thiazolidinediones pioglitazone (Czaja, 2009; Ahmed et al., 2017), dipeptidyl peptidase 4 (DPP-4) inhibitors (Mascolo et al., 2016), glucagon-like peptide 1 (GLP-1) receptor agonists (Drucker, 2018). However, insulin secretagogue agents have to be used with caution because of their significant hypoglycemic risk. Therefore, it is important to develop new natural products that may have potential regulating GMD and IR effects (Jiang et al., 2018; Zhang et al., 2021; Xu J. et al., 2022). This study found that H-HGyp could significantly reduce the fasting blood glucose of HFD mice after 4 weeks of administration, and significantly improve the glucose tolerance and insulin tolerance of HFD-induced IR mice after 12 weeks administration, which could reduce the IR of mice. T2DM is also a concern of obese patients (Smith and Kahn, 2016). It has increased in both developed and developing countries (Yang et al., 2018; Sarma et al., 2021). HFD administration induces GMD and IR, and exhibits clinical and histopathological characteristics similar to those of human GMD and IR, such as weight loss, blood glucose increasing, liver and kidney damage. Therefore, HFD-induced experimental animals are widely accepted model for studying GMD and IR pathogenesis. In this study, HFD induced mice increased body weight in the first 10 weeks and then began to decrease, higher blood glucose, TCHO, TG, LDL-C, insulin, OGTT and GSP of serum. In comparison to the HFD group, HGyp groups suppressed the clinical symptoms of GMD and histological damage to the liver. Particularly H-HGyp shown a similarity to the NFD group, a positive control. These results were consistent with previous studies showing that HGyp improved GMD and IR, suggesting that HGyp effectively suppresses the symptoms of GMD and IR.

Methylglyoxal (MGO) buildup is enhanced by high blood glucose levels. MGO is a highly reactive dicarbonyl molecule that plays a key role in the AGEs production (Nigro et al., 2017; Schalkwijk and Stehouwer, 2020). The glyoxalase system converts approximately 99% of MGO under physiological circumstances (Rabbani and Thornalley, 2014). Glyoxalase I (Glo1), which catalyses the primary detoxification step by converting the spontaneously generated MGO-GSH hemithioacetal to the thioester S-d-lactoylglutathione, acts as the rate-limiting enzyme in the glyoxalase system (Nigro et al., 2017). Its activity is compromised by oxidative stress. Advanced glycation end products (AGEs) are a class of heterogeneous molecules that are increasingly produced in hyperglycemic circumstances, and their levels have been shown to be positive correlation correlated with insulin resistance (IR) in both human and animal studies (Nowotny et al., 2015). The AGEs receptor (RAGE) is a non-specific multiligand pattern recognition receptor that interacts with a broad variety of ligands, is expressed on several cell types, and has a variety of activities (Dong et al., 2022). It was previously understood that ligand binding to RAGE activates NAPDH oxidases, increasing intracellular ROS production and AGE formation. In this study, mice that had been given the HFD experienced prolonged oxidative stress, as evidenced by reduced GLO1 expression and elevated AGE and RAGE levels that were consistent with predictions. In contrast, HGyp, particularly H-HGyp, was able to greatly outperform HFD on these metrics. As was already indicated, HGyp improved GMD by reducing oxidative stress and acting on the AGE-RAGE signaling pathway. In addition, the rate-limiting phase of peripheral glucose consumption is transmembrane glucose transfer mediated by glucose transporter 4 (GLUT4), an insulin-regulated glucose transporter that transports and locates to the plasma membrane for glucose absorption. Studies have shown that an important potential cause of IR is reduced glucose absorption, which is mediated by GLUT4 (Herman et al., 2022). There is also evidence that insulin-stimulated GLUT4 translocation is dependent on PI3K and Akt activation. In this study, GLUT4 expression was concentration-dependent, suggesting that HGyp may increase glucose transport by increasing GLUT4 level, thus improving GMD. However, how HGyp affects glucose transport remains to be further studied.

## 5 Conclusion

In conclusion, HGyp decreased the histological liver damage and signs of GMD. As well as reducing oxidative stress and acting on the AGE-RAGE signaling cascade, HGyp increased the expression of GLUT4 to protect against GMD. Thus, these data imply that HGyp efficiently guards against GMD and may be helpful in the development of treatment approaches or functional goods.

## Data Availability

The datasets presented in this study can be found in online repositories. The names of the repository/repositories and accession number(s) can be found in the article/Supplementary Material.

## Abbreviations

AGEs, advanced glycation end products; Gyp, gypenosides; GMD, glucose metabolic disorders; GSP, glycosylated serum protein; GSH, glutathione; HGyp, gypenosides of heat-processed Gynostemma pentaphyllum; HOMA-IR, homeostasis model assessment of insulin resistance; HFD, high-fat diet; IR, insulin resistance; Met, metformin; MDA, malonic dialdehyde; OGTT, oral glucose tolerance test; SOD, superoxide dismutase.

## References

[B1] Afshin A. Forouzanfar M. H. Reitsma M. B. Sur P. Estep K. Lee A. (2017). Health effects of overweight and obesity in 195 countries over 25 years. N. Engl. J. Med. 377, 13–27. 10.1056/NEJMoa1614362 28604169 PMC5477817

[B2] Ahmed O. A. A. El-Say K. M. Alahdal A. M. (2017). A PLGA-reinforced PEG *in situ* gel formulation for improved sustainability of hypoglycaemic activity of glimepiride in streptozotocin-induced diabetic rats. Sci. Rep. 7, 16384. 10.1038/s41598-017-16728-0 29180715 PMC5703987

[B3] Aschner P. Karuranga S. James S. Simmons D. Basit A. Shaw J. E. (2021). The International Diabetes Federation's guide for diabetes epidemiological studies. Diabetes Res. Clin. Pract. 172, 108630. 10.1016/j.diabres.2020.108630 33347900

[B4] Bharath L. P. Nikolajczyk B. S. (2021). The intersection of metformin and inflammation. Am. J. Physiol. Cell Physiol. 320, C873–C879. 10.1152/ajpcell.00604.2020 33689478 PMC8163577

[B5] Boden G. Homko C. Barrero C. A. Stein T. P. Chen X. Cheung P. (2015). Excessive caloric intake acutely causes oxidative stress, GLUT4 carbonylation, and insulin resistance in healthy men. Sci. Transl. Med. 7, 304re7. 10.1126/scitranslmed.aac4765 PMC5600191 26355033

[B6] Butler A. E. Campbell-Thompson M. Gurlo T. Dawson D. W. Atkinson M. Butler P. C. (2013). Marked expansion of exocrine and endocrine pancreas with incretin therapy in humans with increased exocrine pancreas dysplasia and the potential for glucagon-producing neuroendocrine tumors. Diabetes 62, 2595–2604. 10.2337/db12-1686 23524641 PMC3712065

[B7] Cai W. Ramdas M. Zhu L. Chen X. Striker G. E. Vlassara H. (2012). Oral advanced glycation endproducts (AGEs) promote insulin resistance and diabetes by depleting the antioxidant defenses AGE receptor-1 and sirtuin 1. Proc. Natl. Acad. Sci. U. S. A. 109, 15888–15893. 10.1073/pnas.1205847109 22908267 PMC3465382

[B8] Chadt A. Al-Hasani H. (2020). Glucose transporters in adipose tissue, liver, and skeletal muscle in metabolic health and disease. Pflugers Arch. 472, 1273–1298. 10.1007/s00424-020-02417-x 32591906 PMC7462924

[B9] Czaja M. J. (2009). Pioglitazone: more than just an insulin sensitizer. Hepatology 49, 1427–1430. 10.1002/hep.22983 19402055 PMC2689873

[B10] Davidson M. B. Schriger D. L. (2010). Effect of age and race/ethnicity on HbA1c levels in people without known diabetes mellitus: implications for the diagnosis of diabetes. Diabetes Res. Clin. Pract. 87, 415–421. 10.1016/j.diabres.2009.12.013 20061043

[B11] Dong H. Zhang Y. Huang Y. Deng H. (2022). Pathophysiology of RAGE in inflammatory diseases. Front. Immunol. 13, 931473. 10.3389/fimmu.2022.931473 35967420 PMC9373849

[B12] Drucker D. J. (2018). Mechanisms of action and therapeutic application of glucagon-like peptide-1. Cell Metab. 27, 740–756. 10.1016/j.cmet.2018.03.001 29617641

[B13] Flory J. Lipska K. (2019). Metformin in 2019. Jama 321, 1926–1927. 10.1001/jama.2019.3805 31009043 PMC7552083

[B14] Herman R. Kravos N. A. Jensterle M. Janez A. Dolzan V. (2022). Metformin and insulin resistance: A review of the underlying mechanisms behind changes in GLUT4-mediated glucose transport. Int. J. Mol. Sci. 23, 1264. 10.3390/ijms23031264 35163187 PMC8836112

[B15] Hotamisligil G. S. (2017). Inflammation, metaflammation and immunometabolic disorders. Nature 542, 177–185. 10.1038/nature21363 28179656

[B16] Jiang B. Lv Q. Wan W. Le L. Xu L. Hu K. (2018). Transcriptome analysis reveals the mechanism of the effect of flower tea Coreopsis tinctoria on hepatic insulin resistance. Food Funct. 9, 5607–5620. 10.1039/c8fo00965a 30370909

[B17] Jiashuo W. U. Fangqing Z. Zhuangzhuang L. I. Weiyi J. Yue S. (2022). Integration strategy of network pharmacology in traditional Chinese medicine: a narrative review. J. Tradit. Chin. Med. 42, 479–486. 10.19852/j.cnki.jtcm.20220408.003 35610020 PMC9924699

[B18] Kahn S. E. Cooper M. E. Del Prato S. (2014). Pathophysiology and treatment of type 2 diabetes: perspectives on the past, present, and future. Lancet 383, 1068–1083. 10.1016/S0140-6736(13)62154-6 24315620 PMC4226760

[B19] Kahn S. E. Hull R. L. Utzschneider K. M. (2006). Mechanisms linking obesity to insulin resistance and type 2 diabetes. Nature 444, 840–846. 10.1038/nature05482 17167471

[B20] Kang Q. Yang C. (2020). Oxidative stress and diabetic retinopathy: Molecular mechanisms, pathogenetic role and therapeutic implications. Redox Biol. 37, 101799. 10.1016/j.redox.2020.101799 33248932 PMC7767789

[B21] Kao T. H. Huang S. C. Inbaraj B. S. Chen B. H. (2008). Determination of flavonoids and saponins in Gynostemma pentaphyllum (Thunb.) Makino by liquid chromatography-mass spectrometry. Anal. Chim. Acta 626, 200–211. 10.1016/j.aca.2008.07.049 18790122

[B22] Khan I. Huang G. Li X. A. Liao W. Leong W. K. Xia W. (2019). Mushroom polysaccharides and jiaogulan saponins exert cancer preventive effects by shaping the gut microbiota and microenvironment in Apc(Min/+) mice. Pharmacol. Res. 148, 104448. 10.1016/j.phrs.2019.104448 31499195

[B23] Kim J. H. Han Y. N. (2011). Dammarane-type saponins from Gynostemma pentaphyllum. Phytochemistry 72, 1453–1459. 10.1016/j.phytochem.2011.04.003 21565370

[B24] Lascar N. Brown J. Pattison H. Barnett A. H. Bailey C. J. Bellary S. (2018). Type 2 diabetes in adolescents and young adults. Lancet Diabetes Endocrinol. 6, 69–80. 10.1016/S2213-8587(17)30186-9 28847479

[B25] Lazzaroni E. Ben Nasr M. Loretelli C. Pastore I. Plebani L. Lunati M. E. (2021). Anti-diabetic drugs and weight loss in patients with type 2 diabetes. Pharmacol. Res. 171, 105782. 10.1016/j.phrs.2021.105782 34302978

[B26] Lee S. A. Jo H. K. Im B. O. Kim S. Whang W. K. Ko S. K. (2012). Changes in the contents of prosapogenin in the red ginseng (Panax ginseng) depending on steaming batches. J. Ginseng Res. 36, 102–106. 10.5142/jgr.2012.36.1.102 23717110 PMC3659570

[B27] Lee S. M. (2014). Anti-inflammatory effects of ginsenosides Rg5, Rz1, and Rk1: inhibition of TNF-α-induced NF-κB, COX-2, and iNOS transcriptional expression. Phytother. Res. 28, 1893–1896. 10.1002/ptr.5203 25042112

[B28] Li Y. Lin W. Huang J. Xie Y. Ma W. (2016). Anti-cancer effects of Gynostemma pentaphyllum (Thunb.) Makino (jiaogulan). Chin. Med. 11, 43. 10.1186/s13020-016-0114-9 27708693 PMC5037898

[B29] Liu H. Li X. Duan Y. Xie J. B. Piao X. L. (2021). Mechanism of gypenosides of Gynostemma pentaphyllum inducing apoptosis of renal cell carcinoma by PI3K/AKT/mTOR pathway. J. Ethnopharmacol. 271, 113907. 10.1016/j.jep.2021.113907 33556477

[B30] Liu J. Li Y. Yang P. Wan J. Chang Q. Wang T. T. Y. (2017). Gypenosides reduced the risk of overweight and insulin resistance in C57bl/6J mice through modulating adipose thermogenesis and gut microbiota. J. Agric. Food Chem. 65, 9237–9246. 10.1021/acs.jafc.7b03382 28975783

[B31] Mascolo A. Rafaniello C. Sportiello L. Sessa M. Cimmaruta D. Rossi F. (2016). Dipeptidyl peptidase (DPP)-4 inhibitor-induced arthritis/arthralgia: A review of clinical cases. Drug Saf. 39, 401–407. 10.1007/s40264-016-0399-8 26873369

[B32] Nigro C. Leone A. Raciti G. A. Longo M. Mirra P. Formisano P. (2017). Methylglyoxal-glyoxalase 1 balance: The root of vascular damage. Int. J. Mol. Sci. 18, 188. 10.3390/ijms18010188 28106778 PMC5297820

[B33] Norberg A. Hoa N. K. Liepinsh E. Van Phan D. Thuan N. D. Jörnvall H. (2004). A novel insulin-releasing substance, phanoside, from the plant Gynostemma pentaphyllum. J. Biol. Chem. 279, 41361–41367. 10.1074/jbc.M403435200 15220351

[B34] Nowotny K. Jung T. Höhn A. Weber D. Grune T. (2015). Advanced glycation end products and oxidative stress in type 2 diabetes mellitus. Biomolecules 5, 194–222. 10.3390/biom5010194 25786107 PMC4384119

[B35] Pandey A. Chawla S. Guchhait P. (2015). Type-2 diabetes: Current understanding and future perspectives. IUBMB Life 67, 506–513. 10.1002/iub.1396 26177573

[B36] Park J. Y. Choi P. Kim T. Ko H. Kim H. K. Kang K. S. (2015). Protective effects of processed ginseng and its active ginsenosides on cisplatin-induced nephrotoxicity: *In vitro* and *in vivo* studies. J. Agric. Food Chem. 63, 5964–5969. 10.1021/acs.jafc.5b00782 26050847

[B37] Petersen M. C. Shulman G. I. (2018). Mechanisms of insulin action and insulin resistance. Physiol. Rev. 98, 2133–2223. 10.1152/physrev.00063.2017 30067154 PMC6170977

[B38] Phillips P. J. (2012). Oral glucose tolerance testing. Aust. Fam. Physician 41, 391–393. 22675678

[B39] Rabbani N. Thornalley P. J. (2014). Dicarbonyl proteome and genome damage in metabolic and vascular disease. Biochem. Soc. Trans. 42, 425–432. 10.1042/BST20140018 24646255

[B40] Rachdaoui N. (2020). Insulin: The friend and the foe in the development of type 2 diabetes mellitus. Int. J. Mol. Sci. 21, 1770. 10.3390/ijms21051770 32150819 PMC7084909

[B41] Ren D. Zhao Y. Zheng Q. Alim A. Yang X. (2019). Immunomodulatory effects of an acidic polysaccharide fraction from herbal Gynostemma pentaphyllum tea in RAW264.7 cells. Food Funct. 10, 2186–2197. 10.1039/c9fo00219g 30942219

[B42] Sakyi S. A. Laing E. F. Mantey R. Kwarteng A. Owiredu E. W. Dadzie R. E. (2021). Profiling immuno-metabolic mediators of vitamin B12 deficiency among metformin-treated type 2 diabetic patients in Ghana. PLoS One 16, e0249325. 10.1371/journal.pone.0249325 33784336 PMC8009370

[B43] Salpeter S. R. Greyber E. Pasternak G. A. Salpeter Posthumous E. E. (2010). Risk of fatal and nonfatal lactic acidosis with metformin use in type 2 diabetes mellitus. Cochrane Database Syst. Rev. 2010, CD002967. 10.1002/14651858.CD002967 20393934 PMC7138050

[B44] Saltiel A. R. (2021). Insulin signaling in health and disease. J. Clin. Invest. 131, e142241. 10.1172/JCI142241 33393497 PMC7773347

[B45] Sarma S. Sockalingam S. Dash S. (2021). Obesity as a multisystem disease: Trends in obesity rates and obesity-related complications. Diabetes Obes. Metab. 23 (1), 3–16. 10.1111/dom.14290 33621415

[B46] Schalkwijk C. G. Stehouwer C. D. A. (2020). Methylglyoxal, a highly reactive dicarbonyl compound, in diabetes, its vascular complications, and other age-related diseases. Physiol. Rev. 100, 407–461. 10.1152/physrev.00001.2019 31539311

[B47] Shin B. K. Kwon S. W. Park J. H. (2015). Chemical diversity of ginseng saponins from Panax ginseng. J. Ginseng Res. 39, 287–298. 10.1016/j.jgr.2014.12.005 26869820 PMC4593792

[B48] Smith U. Kahn B. B. (2016). Adipose tissue regulates insulin sensitivity: role of adipogenesis, de novo lipogenesis and novel lipids. J. Intern Med. 280, 465–475. 10.1111/joim.12540 27699898 PMC5218584

[B49] Song J. X. An J. R. Chen Q. Yang X. Y. Jia C. L. Xu S. (2022). Liraglutide attenuates hepatic iron levels and ferroptosis in db/db mice. Bioengineered 13, 8334–8348. 10.1080/21655979.2022.2051858 35311455 PMC9161873

[B50] Tang Q. Li X. Song P. Xu L. (2015). Optimal cut-off values for the homeostasis model assessment of insulin resistance (HOMA-IR) and pre-diabetes screening: Developments in research and prospects for the future. Drug Discov. Ther. 9, 380–385. 10.5582/ddt.2015.01207 26781921

[B51] Uribarri J. Cai W. Ramdas M. Goodman S. Pyzik R. Chen X. (2011). Restriction of advanced glycation end products improves insulin resistance in human type 2 diabetes: potential role of AGER1 and SIRT1. Diabetes Care 34, 1610–1616. 10.2337/dc11-0091 21709297 PMC3120204

[B52] Vlassara H. Uribarri J. (2014). Advanced glycation end products (AGE) and diabetes: cause, effect, or both? Curr. Diab Rep. 14, 453. 10.1007/s11892-013-0453-1 24292971 PMC3903318

[B53] Wallace T. M. Levy J. C. Matthews D. R. (2004). Use and abuse of HOMA modeling. Diabetes Care 27, 1487–1495. 10.2337/diacare.27.6.1487 15161807

[B54] Wang J. Yang J. L. Zhou P. P. Meng X. H. Shi Y. P. (2017). Further new gypenosides from jiaogulan (Gynostemma pentaphyllum). J. Agric. Food Chem. 65, 5926–5934. 10.1021/acs.jafc.7b01477 28662582

[B55] Xia X. Xu J. Wang X. Wang H. Lin Z. Shao K. (2020). Jiaogulan tea (Gpostemma pentaphyllum) potentiates the antidiabetic effect of white tea via the AMPK and PI3K pathways in C57BL/6 mice. Food Funct. 11, 4339–4355. 10.1039/d0fo00395f 32369096

[B56] Xie P. Guo M. Xie J. B. Xiao M. Y. Qi Y. S. Duan Y. (2022). Effects of heat-processed Gynostemma pentaphyllum on high-fat diet-fed mice of obesity and functional analysis on network pharmacology and molecular docking strategy. J. Ethnopharmacol. 294, 115335. 10.1016/j.jep.2022.115335 35513215

[B57] Xie P. Xie J. B. Xiao M. Y. Guo M. Qi Y. S. Li F. F. (2023). Liver lipidomics analysis reveals the anti-obesity and lipid-lowering effects of gypnosides from heat-processed Gynostemma pentaphyllum in high-fat diet fed mice. Phytomedicine 115, 154834. 10.1016/j.phymed.2023.154834 37094422

[B58] Xie Z. Huang H. Zhao Y. Shi H. Wang S. Wang T. T. (2012). Chemical composition and anti-proliferative and anti-inflammatory effects of the leaf and whole-plant samples of diploid and tetraploid Gynostemma pentaphyllum (Thunb.) Makino. Food Chem. 132, 125–133. 10.1016/j.foodchem.2011.10.043 26434271

[B59] Xing S. F. Liu L. H. Zu M. L. Lin M. Zhai X. F. Piao X. L. (2019). Inhibitory effect of damulin B from Gynostemma pentaphyllum on human lung cancer cells. Planta Med. 85, 394–405. 10.1055/a-0810-7738 30562828

[B60] Xu F. Zhu Y. Lu M. Qin L. Zhao D. Ren T. (2022a). Effects of hydroxy-alpha-sanshool on intestinal metabolism in insulin-resistant mice. Foods 11, 2040. 10.3390/foods11142040 35885283 PMC9322383

[B61] Xu J. Dong J. Ding H. Wang B. Wang Y. Qiu Z. (2022b). Ginsenoside compound K inhibits obesity-induced insulin resistance by regulation of macrophage recruitment and polarization *via* activating PPARγ. Food Funct. 13, 3561–3571. 10.1039/d1fo04273d 35260867

[B62] Yamagishi S. I. (2019). Role of advanced glycation endproduct (AGE)-Receptor for advanced glycation endproduct (RAGE) Axis in cardiovascular disease and its therapeutic intervention. Circ. J. 83, 1822–1828. 10.1253/circj.CJ-19-0618 31366777

[B63] Yang Q. Vijayakumar A. Kahn B. B. (2018). Metabolites as regulators of insulin sensitivity and metabolism. Nat. Rev. Mol. Cell Biol. 19, 654–672. 10.1038/s41580-018-0044-8 30104701 PMC6380503

[B64] Zhang F. Xu S. Tang L. Pan X. Tong N. (2020a). Acarbose with comparable glucose-lowering but superior weight-loss efficacy to dipeptidyl peptidase-4 inhibitors: A systematic review and network meta-analysis of randomized controlled trials. Front. Endocrinol. (Lausanne) 11, 288. 10.3389/fendo.2020.00288 32582019 PMC7291873

[B65] Zhang H. Tang L. Zhang Q. Xu Q. (2018a). Gypenosides inhibit AGEs induced TGF- beta 1 and PDGF expression in human glomerular mesangial cells. Acta Med. Sin. 31, 50–53.

[B66] Zhang P. Li T. Wu X. Nice E. C. Huang C. Zhang Y. (2020b). Oxidative stress and diabetes: antioxidative strategies. Front. Med. 14, 583–600. 10.1007/s11684-019-0729-1 32248333

[B67] Zhang Y. Kishi H. Kobayashi S. (2018b). Add-on therapy with traditional Chinese medicine: An efficacious approach for lipid metabolism disorders. Pharmacol. Res. 134, 200–211. 10.1016/j.phrs.2018.06.004 29935947

[B68] Zhang Y. Liu J. Mao G. Zuo J. Li S. Yang Y. (2021). Sargassum fusiforme fucoidan alleviates diet-induced insulin resistance by inhibiting colon-derived ceramide biosynthesis. Food Funct. 12, 8440–8453. 10.1039/d1fo01272j 34374401

[B69] Zhao Z. Zhao C. Zhang X. H. Zheng F. Cai W. Vlassara H. (2009). Advanced glycation end products inhibit glucose-stimulated insulin secretion through nitric oxide-dependent inhibition of cytochrome c oxidase and adenosine triphosphate synthesis. Endocrinology 150, 2569–2576. 10.1210/en.2008-1342 19246537 PMC2689792

[B70] Zu M. L. Duan Y. Xie J. B. Qi Y. S. Xie P. Borjigidai A. (2021). Gypenoside LI arrests the cell cycle of breast cancer in G0/G1 phase by down-regulating E2F1. J. Ethnopharmacol. 273, 114017. 10.1016/j.jep.2021.114017 33716078

