# Supplementary material for: Protective effect of heat-processed Gynostemma pentaphyllum on high fat diet-induced glucose metabolic disorders mice
Source: Front Pharmacol. 2023 Sep 25;14:1215150. doi: 10.3389/fphar.2023.1215150 (PMC10563512; doi:10.3389/fphar.2023.1215150)
Supplement: Supplementary file 1 [file DataSheet1.docx]

**Table S1 Qualitative results of gypenosides by UPLC-Q-TOF-MS/MS**

| NO. | RT  (min) | Formula | [M-H]^-^ | | Error  (ppm) | [M+HCOO]- | MS2 | Identification | Reference |
| --- | --- | --- | --- | --- | --- | --- | --- | --- | --- |
|  |  |  | Predictived | Measured |  |  |  |  |  |
| 1^b^ | 2.045 | C_48_H_84_O_21_ | 995.5432 | 995.5456 | 2.4 | 1041.5486 | 833.4951, 815.4831, 653.4268, 491.3704 | Unknown | / |
| 2 | 2.735 | C_48_H_84_O_21_ | 995.5432 | 995.5458 | 2.6 | 1041.5476 | 833.4930, 815.4833, 653.4270, 491.3799 | Unknown | / |
| 3 | 4.520 | C_53_H_90_O_24_ | 1109.5749 | 1109.5752 | 0.2 | 1155.5796 | 977.5306, 815.4866, 797.4737, 635.4141 | Gypenoside J5; Gypenoside J6 | Xing, 2019 |
|  |  |  |  |  |  |  |  | Gypenoside LXI; Gypenoside LXVIII; Compd 7; Compd 3 | Kao et al., 2008 |
| 4^b^ | 5.148 | C_48_H_82_O_20_ | 977.5327 | 977.5343 | 1.7 | 1023.5380 | 815.4825, 797.4714, 653.4260, 635.4184 | Gypenoside J2; Gypenoside J1 | Xing et al., 2020 |
| 5 | 5.234 | C_53_H_90_O_24_ | 1109.5749 | 1109.5755 | 0.5 | 1155.5822 | 977.5393, 815.4821, 797.4707, 635.4225 | Gypenoside J5; Gypenoside J6 | Xing, 2019 |
|  |  |  |  |  |  |  |  | Gypenoside LXI; Gypenoside LXVIII; Compd 7; Compd 3 | Kao et al., 2008 |
| 6 | 5.661 | C_53_H_90_O_25_ | 1125.5698 | 1125.5710 | 1.0 | 1171.5707 | 831.4737, 813.4675, 651.4117 | Gypenoside J4 | Xing, 2019 |
|  |  |  |  |  |  |  |  | Compd 2 | Liu et al., 2005 |
| 7 | 5.814 | C_48_H_82_O_20_ | 977.5327 | 977.5349 | 2.3 | 1023.5562 | 815.4811, 797.4724, 653.4239, 635.4206 | Gypenoside J2; Gypenoside J1 | Xing et al., 2020 |
| 8 | 6.041 | C_53_H_90_O_25_ | 1125.5698 | 1125.5714 | 1.4 | 1171.5945 | 945.5092, 831.4783, 813.4614, 651.4069, 633.3997 | Gypenoside J4 | Xing, 2019 |
|  |  |  |  |  |  |  |  | Compd 2 | Liu et al., 2005 |
| 9 | 6.366 | C_48_H_82_O_21_ | 993.5276 | 993.5298 | 2.2 | 1039.5349 | 831.4764, 813.4682, 651.4116, 489.3583 | Unknown | / |
| 10^b^ | 6.534 | C_48_H_82_O_20_ | 977.5327 | 977.5353 | 2.7 | 1023.5637 | 815.4809, 797.4715, 653.4284, 635.4154 | Gypenoside J2; Gypenoside J1 | Xing et al., 2020 |
| 11 | 6.647 | C_48_H_82_O_21_ | 993.5276 | 993.5301 | 2.5 | 1039.5314 | 975.5232, 813.4700, 651.4134, 633.4009, 489.3588 | Unknown | / |
| 12 | 7.224 | C_48_H_80_O_20_ | 975.5170 | 975.5189 | 1.9 | 1021.5106 | 813.4677, 795.4599, 633.4009, 471.3517 | Unknown | Wang et al., 2020 |
| 13 | 7.473 | C_53_H_90_O_24_ | 1109.5749 | 1109.5780 | 2.8 | 1155.5780 | 977.5338, 797.4726, 635.4149 | Gypenoside J5; Gypenoside J6 | Xing, 2019 |
|  |  |  |  |  |  |  |  | Gypenoside LXI; Gypenoside LXVIII; Compd 7; Compd 3 | Kao et al., 2008 |
| 14 | 7.631 | C_48_H_82_O_21_ | 993.5276 | 993.5308 | 3.2 | 1039.5334 | 831.4762, 813.4648, 651.4149, 633.4028, 489.3564 | Unknown | / |
| 15 | 7.841 | C_53_H_90_O_24_ | 1109.5749 | 1109.5746 | -0.3 | 1155.5764 | 977.5422, 815.4902, 797.4726, 635.4203 | Gypenoside J5; Gypenoside J6 | Xing, 2019 |
|  |  |  |  |  |  |  |  | Gypenoside LXI; Gypenoside LXVIII; Compd 7; Compd 3 | Kao et al., 2008 |
| 16 | 8.069 | C_42_H_72_O_15_ | 815.4798 | 815.4807 | 1.0 | 861.4734 | 653.4266, 607.3475, 491.3754, 445.3017 | Gypenoside GC5 | Wang et al., 2020 |
|  |  |  |  |  |  |  |  | Yunnangypenoside A; Yunnangypenoside B | Yin et al., 2020 |
| 17^b^ | 8.261 | C_50_H_84_O_21_ | 1019.5432 | 1019.5446 | 1.3 | 1065.5483 | 977.5330, 959.5252, 839.4824, 797.4693, 779.4610, 635.4146 | Acetyl-Gypenoside J1;  Acetyl-Gypenoside J2 | / |
| 18 | 8.359 | C_48_H_80_O_20_ | 975.5170 | 975.5184 | 1.4 | 1021.5478 | 813.4652, 795.4605, 633.4038 | Unknown | Wang et al., 2020 |
| 19 | 8.785 | C_50_H_84_O_21_ | 1019.5432 | 1019.5447 | 1.4 | 1065.5489 | 977.5367, 959.5251, 779.4575 | Acetyl-Gypenoside J1;  Acetyl-Gypenoside J2 | / |
| 20 | 8.863 | C_53_H_90_O_24_ | 1109.5749 | 1109.5756 | 0.6 | 1155.5807 | 977.5400, 815.4829, 797.4788, 635.4097 | Gypenoside J5; Gypenoside J6 | Xing, 2019 |
|  |  |  |  |  |  |  |  | Gypenoside LXI; Gypenoside LXVIII; Compd 7; Compd 3 | Kao et al., 2008 |
| 21 | 9.098 | C_48_H_80_O_19_ | 959.5221 | 959.5232 | 1.1 | 1005.5262 | 797.4806, 779.4595, 635.4210 | Gypenoside XXIV | Wang et al., 2020 |
| 22 | 9.328 | C_50_H_84_O_21_ | 1019.5432 | 1019.5425 | -0.7 | 1065.5600 | 977.5352, 959.5234, 797.4729, 635.4184 | Acetyl -Gypenoside J1;  Acetyl -Gypenoside J2 | / |
| 23 | 9.346 | C_42_H_72_O_15_ | 815.4798 | 815.4809 | 1.3 | 861.4890 | 653.4275, 491.3732 | Gypenoside GC5 | Wang et al., 2020 |
|  |  |  |  |  |  |  |  | Yunnangypenoside A; Yunnangypenoside B | Yin et al., 2020 |
| 24^b^ | 10.030 | C_42_H_72_O_15_ | 815.4798 | 815.4810 | 1.4 | 861.4833 | 653.4285,491.3748 | Gypenoside GC5 | Wang et al., 2020 |
|  |  |  |  |  |  |  |  | Yunnangypenoside A; Yunnangypenoside B | Yin et al., 2020 |
| 25 | 10.901 | C_42_H_70_O_15_ | 813.4642 | 813.4656 | 1.7 | 859.4696 | 651.4115, 489.3582 | Gypenoside GD1 | Wang et al., 2020 |
| 26^a,b^ | 11.507 | C_53_H_90_O_23_ | 1093.5800 | 1093.5827 | 2.5 | 1139.5825 | 931.5292, 799.4860, 637.4314, 475.3789 | Gypenoside LVI | Duan et al., 2021 |
| 27 | 11.833 | C_52_H_88_O_22_ | 1063.5694 | 1063.5704 | 0.9 | 1109.5768 | 901.5138, 769.4666, 637.4361, 475.3805 | Unknown | Wang et al., 2020 |
| 28^b^ | 12.005 | C_42_H_72_O_15_ | 815.4798 | 815.4811 | 1.5 | 861.4826 | 653.4285, 491.3732 | Gypenoside GC5 | Wang et al., 2020 |
|  |  |  |  |  |  |  |  | Yunnangypenoside A; Yunnangypenoside B | Yin et al., 2020 |
| 29 | 12.114 | C_48_H_80_O_19_ | 959.5221 | 959.5218 | -0.3 | 1005.5267 | 797.4709, 635.4153 | Gypenoside XXIV | Wang et al., 2020 |
| 30 | 12.252 | C_53_H_90_O_23_ | 1093.5800 | 1093.5801 | 0.1 | 1139.5775 | 961.5417, 931.5285, 799.4819, 637.4341, 475.3856 | Gypenoside LXVII; Gypenoside XXII; Gypenoside LXXI | Wang et al., 2020 |
| 31^a,b^ | 12.325 | C_48_H_82_O_19_ | 961.5378 | 961.5385 | 0.8 | 1007.5415 | 799.4873, 637.4335, 475.3784 | Gypenoside XLVI | Duan et al., 2021 |
| 32 | 12.434 | C_42_H_72_O_15_ | 815.4798 | 815.4808 | 1.2 | 861.4833 | 653.4285, 491.3747 | Gypenoside GC5 | Wang et al., 2020 |
|  |  |  |  |  |  |  |  | Yunnangypenoside A; Yunnangypenoside B | Yin et al., 2020 |
| 33^b^ | 12.564 | C_53_H_90_O_22_ | 1077.5851 | 1077.5859 | 0.7 | 1123.5888 | 945.5431, 915.5340, 783.4931, 621.4388, 459.3877 | Ginsenoside Rc; Ginsenoside Rb2; Gypenoside LXIII; Gypenoside LV | Wang et al., 2020 |
| 34 | 12.660 | C_47_H_80_O_18_ | 931.5272 | 931.5282 | 1.1 | 977.5296 | 799.4834, 769.4771, 637.4382, 607.4239, 475.3794 | Gypenoside LVII | Xing et al., 2020 |
|  |  |  |  |  |  |  |  | Notoginsenoside Ft3; Notoginsenoside Ft5 | Wang et al., 2020 |
| 35 | 12.798 | C_42_H_72_O_15_ | 815.4798 | 815.4837 | 4.7 | 861.4809 | 653.4253, 491.3706 | Gypenoside GC5 | Wang et al., 2020 |
|  |  |  |  |  |  |  |  | Yunnangypenoside A; Yunnangypenoside B | Yin et al., 2020 |
| 36^b^ | 13.170 | C_55_H_92_O_24_ | 1135.5906 | 1135.5912 | 0.5 | 1181.5878 | 1093.5885, 1075.5769, 931.5336, 799.4847, 637.4369, 475.3813 | Acetyl -gypenoside LVI | / |
| 37 | 13.225 | C_42_H_70_O_14_ | 797.4693 | 797.4700 | 0.9 | 843.5024 | 635.4163, 473.3649 | Gypenoside XL; Gypenoside XXXIII | Wang et al., 2020 |
| 38^a,b^ | 13.440 | C_48_H_82_O_18_ | 945.5428 | 945.5439 | 1.1 | 991.5463 | 783.4930, 621.4365, 459.3822 | Ginsenoside Rd | Duan et al., 2021 |
| 39 | 13.749 | C_47_H_80_O_17_ | 915.5323 | 915.5316 | -0.7 | 961.5344 | 783.4934, 621.4401 | Gypenoside LV | Wang et al., 2020 |
| 40^b^ | 14.131 | C_47_H_80_O_17_ | 961.5378 | 961.5371 | -0.7 |  | 915.5333, 753.4785, 591.4248, 459.3821 | Unknown | / |
| 41^b^ | 14.131 | C_50_H_84_O_20_ | 1003.5483 | 1003.5488 | 0.5 | 1049.5515 | 961.5401, 799.4851, 637.4314, 475.3775 | Compd 2 | Hung et al., 2010 |
| 42 | 14.222 | C_42_H_72_O_15_ | 815.4798 | 815.4805 | 0.8 | 861.4838 | 653.4271, 491.3741 | Gypenoside GC5 | Wang et al., 2020 |
|  |  |  |  |  |  |  |  | Yunnangypenoside A; Yunnangypenoside B | Yin et al., 2020 |
| 43 | 14.240 | C_55_H_92_O_23_ | 1119.5957 | 1119.5961 | 0.4 | 1165.5978 | 1077.5840, 915.5303, 783.4904, 621.4311 | Unknown | / |
| 44 | 14.333 | C_42_H_70_O_15_ | 813.4642 | 813.4655 | 1.6 | 859.4705 | 651.4141, 471.3468 | Gypenoside GD1 isomer | Wang et al., 2020 |
| 45^b^ | 14.928 | C_47_H_80_O_18_ | 931.5272 | 931.5311 | 4.2 | 977.5311 | 799.4885, 769.4756, 637.4329, 475.3776 | Gypenoside LVII | Xing et al., 2020 |
|  |  |  |  |  |  |  |  | Notoginsenoside Ft3; Notoginsenoside Ft5 | Wang et al., 2020 |
| 46 | 15.106 | C_42_H_70_O_14_ | 797.4693 | 797.4709 | 2.0 | 843.5003 | 635.4174, 473.3643 | Gypenoside XL; Gypenoside XXXIII | Wang et al., 2020 |
| 47 | 15.320 | C_50_H_84_O_19_ | 987.5534 | 987.5548 | 1.4 | 1033.5578 | 945.5431, 783.4885, 621.4441 | Acetyl -ginsenoside Rd | / |
| 48 | 15.534 | C_42_H_70_O_14_ | 797.4693 | 797.4697 | 0.5 | 843.4849 | 635.4167, 473.3624 | Gypenoside XL; Gypenoside XXXIII | Wang et al., 2020 |
| 49 | 15.974 | C_42_H_70_O_14_ | 797.4693 | 797.4703 | 1.3 | 843.4808 | 635.4164, 473.3626 | Gypenoside XL; Gypenoside XXXIII | Wang et al., 2020 |
| 50^a,b^ | 17.080 | C_42_H_72_O_14_ | 799.4849 | 799.4856 | 0.8 | 845.4887 | 637.4331, 475.3787 | Gypenoside L | Duan et al., 2021 |
| 51^a,b^ | 17.505 | C_42_H_72_O_14_ | 799.4849 | 799.4863 | 1.7 | 845.4881 | 637.4337, 475.3786 | Gypenoside LI | Duan et al., 2021 |
| 52^b^ | 17.749 | C_41_H_70_O_13_ | 769.4744 | 769.4756 | 1.6 | 815.4777 | 607.4225, 475.3780 | Gypenoside LXXVII; Gypenoside LXVI; Gypenoside XXI; Gynoside A; Gynoside C | Wang et al., 2020 |
| 53 | 18.221 | C_42_H_72_O_13_ | 783.4900 | 783.4909 | 1.1 | 829.4924 | 621.4394, 459.3864 | Gypenoside TN2; Gypenoside LXVI; Gypenoside LXXV; Gypenoside XXVII; Gypenoside XLI; Gypenoside XII; Gypenoside LXXIX | Wang et al., 2020 |
| 54 | 18.356 | C_41_H_70_O_13_ | 769.4744 | 769.4764 | 2.6 | 815.4803 | 637.4308, 475.3768 | Gypenoside LXXVII; Gypenoside LXVI; Gypenoside XXI; Gynoside A; Gynoside C | Wang et al., 2020 |
| 55^a,b^ | 18.795 | C_42_H_72_O_13_ | 783.4900 | 783.4905 | 0.6 | 829.4936 | 621.4377, 459.3835 | Ginsenoside Rg3-S | Duan et al., 2021 |
| 56^a,b^ | 19.176 | C_42_H_72_O_13_ | 783.4900 | 783.4908 | 1.0 | 829.4939 | 621.4375, 459.3829 | Ginsenoside Rg3-R | Duan et al., 2021 |
| 57^b^ | 19.213 | C_44_H_74_O_15_ | 841.4955 | 841.4960 | 0.6 | 887.4988 | 799.4893, 637.4336, 475.3802 | Acetyl -gypenoside L | / |
| 58 | 19.613 | C_44_H_74_O_15_ | 841.4955 | 841.4966 | 1.3 | 887.5001 | 799.4877, 637.4328, 475.3778 | Acetyl -gypenoside LI | / |
| 59 | 19.887 | C_41_H_70_O_12_ | 753.4795 | 753.4797 | 0.3 | 799.4840 | 591.4256, 459.3848 | Gypenoside XIII; Gypenoside LXXVIII | Wang et al., 2020 |
| 60 | 20.392 | C_41_H_70_O_12_ | 753.4795 | 753.4798 | 0.5 | 799.4842 | 621.4355 | Gypenoside XIII; Gypenoside LXXVIII | Wang et al., 2020 |
| 61^a,b^ | 20.756 | C_42_H_70_O_13_ | 781.4744 | 781.4753 | 1.2 | 827.4779 | 619.4237, 457.3701 | Damulin B | Duan et al., 2021 |
| 62 | 20.998 | C_41_H_68_O_12_ | 751.4638 | 751.4629 | -1.2 | 797.4683 | 619.4197, 457.3667 | Gypenoside XXIX | Wang et al., 2020 |
| 63^a,b^ | 21.035 | C_42_H_70_O_13_ | 781.4744 | 781.4755 | 1.5 | 827.4796 | 619.4231, 457.3670 | Damulin A | Duan et al., 2021 |
| 64 | 21.736 | C_41_H_68_O_12_ | 751.4638 | 751.4646 | 1.1 | 797.4677 | 589.4122, 457.3674 | Unknown | / |
| 65 | 21.837 | C_36_H_62_O_9_ | 637.4321 | 637.4321 | 0.0 | 683.4370 | 475.3795 | Gypenoside Jh1 | Xing et al., 2016 |
| 66 | 22.157 | C_41_H_68_O_12_ | 751.4638 | 751.4637 | -0.1 | 797.4746 | 589.4091 | Unknown | / |
| 67 | 22.995 | C_44_H_72_O_14_ | 823.4849 | 823.4864 | 1.8 | 869.4895 | 781.4753, 619.4213, 457.3680 | Damulin D | Piao et al., 2014 |
| 68^a,b^ | 23.142 | C_42_H_70_O_12_ | 765.4795 | 765.4812 | 2.3 | 811.4845 | 603.4271, 441.3715 | Ginsenoside Rk1 | Yang, 2021 |
| 69 | 23.431 | C_44_H_72_O_14_ | 823.4849 | 823.4863 | 1.7 | 869.4897 | 781.4764, 619.4216, 457.3675 | Damulin C | Piao et al., 2014 |
| 70^a,b^ | 23.582 | C_42_H_70_O_12_ | 765.4795 | 765.4802 | 1.0 | 811.4831 | 603.4287 | Ginsenoside Rg5 | Yang, 2021 |
| 71 | 25.673 | C_44_H_72_O_13_ | 807.4900 | 807.4904 | 0.5 | 853.4922 | 765.4793, 603.4307 | Unknown | / |
| 72 | 26.247 | C_44_H_72_O_13_ | 807.4900 | 807.4908 | 1.0 | 853.4914 | 765.4800, 603.4277 | Unknown | / |
| 73 | 27.133 | C_42_H_70_O_12_ | 765.4795 | 765.4805 | 1.4 | 811.4880 | 603.4265, 441.3714 | Unknown | Wang et al., 2020 |

The substances with ^a^ in the table have been compared with the reference substance.

The substances with ^b^ in the table were serum migrant compounds.

Xing S.F., 2019. Studies on saponins from Zhuang-medicine "Gocaekmbaw" (Gynostemma pentaphyllum) and their inhibitory effect on NSCLC. [D]. Minzu University of China.

Kao T.H., Huang, S.C., Inbaraj, B.S.,Chen, B.H., 2008. Determination of flavonoids and saponins in Gynostemma pentaphyllum (Thunb.) Makino by liquid chromatography-mass spectrometry. Anal Chim Acta. 626 (2), 200-11. <http://doi.org/10.1016/j.aca.2008.07.049>.

Xing S.F., Lin, M., Wang, Y.R., Chang, T., Cui, W.Y.,Piao, X.L., 2020. Novel dammarane-type saponins from Gynostemma pentaphyllum and their neuroprotective effect. Nat Prod Res. 34 (5), 651-658. <http://doi.org/10.1080/14786419.2018.1495638>.

Liu X., Ye, W., Mo, Z., Yu, B., Wu, H., Zhao, S., Che, C.,Hsiao, W.L., 2005. Three dammarane-type saponins from Gynostemma pentaphyllum. Planta Med. 71 (9), 880-4. <http://doi.org/10.1055/s-2005-871256>.

Wang X., Li, D., Guo, X., Zhang, Q., Liao, X., Cao, Z., Liu, L.,Yang, P., 2020. ComMS(n)DB-An Automatable Strategy to Identify Compounds from MS Data Sets (Identification of Gypenosides as an Example). J Agric Food Chem. 68 (41), 11368-11388. <http://doi.org/10.1021/acs.jafc.0c03693>.

Yin M., Zhang, J., Wang, L., Li, F., Li, Z., Xiang, W., Bie, S., Wang, C.,Li, Z., 2020. Ten New Dammarane-Type Saponins with Hypolipidemia Activity from a Functional Herbal Tea-Gynostemma pentaphyllum. Molecules. 25 (16), <http://doi.org/10.3390/molecules25163737>.

Hung T.M., Thu, C.V., Cuong, T.D., Hung, N.P., Kwack, S.J., Huh, J.I., Min, B.S., Choi, J.S., Lee, H.K.,Bae, K., 2010. Dammarane-type glycosides from Gynostemma pentaphyllum and their effects on IL-4-induced eotaxin expression in human bronchial epithelial cells. J Nat Prod. 73 (2), 192-6. <http://doi.org/10.1021/np9006712>.

Duan Y., Yang, J., Xie, J.B., Xie, P., Qi, Y.S., Zhao, M.T.,Piao, X.L., 2021. [Simultaneous quantitative analysis of nine saponins in Gynostemma pentaphyllum before and after heat processing based on UPLC-Q-Trap-MS]. Zhongguo Zhong Yao Za Zhi. 46 (20), 5314-5319. <http://doi.org/10.19540/j.cnki.cjcmm.20210702.201>.

Xing S.F., Jang, M., Wang, Y.R.,Piao, X.L., 2016. A new dammarane-type saponin from Gynostemma pentaphyllum induces apoptosis in A549 human lung carcinoma cells. Bioorg Med Chem Lett. 26 (7), 1754-9. <http://doi.org/10.1016/j.bmcl.2016.02.046>.

Piao X.L., Xing, S.F., Lou, C.X.,Chen, D.J., 2014. Novel dammarane saponins from Gynostemma pentaphyllum and their cytotoxic activities against HepG2 cells. Bioorg Med Chem Lett. 24 (20), 4831-3. <http://doi.org/10.1016/j.bmcl.2014.08.059>.

Yang C., 2021. Chemical constituents from Gynostemma pentaphyllum and their inhibitory effect on SGC-7901 cells. [D]. Minzu University of China.

**Table S2 Information of anti-GMD targets of HGyp**

| **Component** | **Target** | **Component** | **Target** | **Component** | **Target** | **Component** | **Target** |
| --- | --- | --- | --- | --- | --- | --- | --- |
|  |  |  |  |  |  |  |  |
| Compd 2 | PSEN1 | Compd 2 | NR3C1 | Damulin A | PTPN1 | Damulin B | IL2 |
| Compd 2 | PSEN2 | Compd 2 | TRPV4 | Damulin A | PPARG | Damulin B | HSD11B2 |
| Compd 2 | FGF2 | Compd 2 | SLC2A1 | Damulin A | ALDH2 | Damulin B | SLC5A2 |
| Compd 2 | HTR2B | Compd 2 | HMGCR | Damulin A | F2 | Damulin B | SLC5A1 |
| Compd 2 | ADRA2A | Compd 2 | ABCB11 | Damulin A | HSD11B1 | Damulin B | LGALS3 |
| Compd 2 | DRD1 | Compd 2 | PFKFB3 | Damulin A | MMP1 | Damulin B | CASP3 |
| Compd 2 | HTR2C | Compd 2 | MMP1 | Damulin A | MME | Damulin B | MMP2 |
| Compd 2 | CYP2D6 | Damulin A | FGF2 | Damulin A | MTOR | Damulin B | ACE |
| Compd 2 | IL2 | Damulin A | HTR2B | Damulin A | PIK3CA | Damulin B | F7 |
| Compd 2 | HSD11B2 | Damulin A | DRD1 | Damulin A | PIK3CG | Damulin B | PTPN1 |
| Compd 2 | LGALS3 | Damulin A | CYP2D6 | Damulin A | EGFR | Damulin B | PPARG |
| Compd 2 | IGF1R | Damulin A | IL2 | Damulin A | MAPK14 | Damulin B | ADRB2 |
| Compd 2 | REN | Damulin A | HSD11B2 | Damulin A | F9 | Damulin B | SIRT2 |
| Compd 2 | DRD2 | Damulin A | SLC5A2 | Damulin A | ICAM1 | Damulin B | MMP1 |
| Compd 2 | DRD3 | Damulin A | SLC5A1 | Damulin A | SELE | Damulin B | MME |
| Compd 2 | PPP2CA | Damulin A | IGF1R | Damulin A | CXCR2 | Damulin B | PIK3CA |
| Compd 2 | ADRB2 | Damulin A | CASP3 | Damulin A | MAP2K1 | Damulin B | PIK3CG |
| Compd 2 | F2 | Damulin A | P2RY12 | Damulin A | GBA2 | Damulin B | EGFR |
| Compd 2 | HSD11B1 | Damulin A | PTPN22 | Damulin A | UGCG | Damulin B | MAPK8 |
| Compd 2 | JUN | Damulin A | ITGB3 | Damulin A | ALOX5 | Damulin B | MAPK14 |
| Compd 2 | PRKCD | Damulin A | REN | Damulin A | FGFR2 | Damulin B | ICAM1 |
| Compd 2 | PTGS2 | Damulin A | MMP2 | Damulin B | FGF2 | Damulin B | MAP2K1 |
| Compd 2 | ABCB1 | Damulin A | ACE | Damulin B | HTR2B | Damulin B | ADRB3 |
| Compd 2 | AR | Damulin A | F7 | Damulin B | CYP2D6 | Damulin B | SERPINE1 |
| Damulin B | PIN1 | Ginsenoside Rd | NR3C1 | Ginsenoside Rg3-R | ACE | Ginsenoside Rg3-S | F7 |
| Damulin B | IGFBP3 | Ginsenoside Rd | MTOR | Ginsenoside Rg3-R | AGTR1 | Ginsenoside Rg3-S | PTPN1 |
| Damulin B | DGAT1 | Ginsenoside Rd | PIK3CG | Ginsenoside Rg3-R | F7 | Ginsenoside Rg3-S | PPARG |
| Ginsenoside Rd | PSEN1 | Ginsenoside Rd | MAP2K1 | Ginsenoside Rg3-R | PTPN1 | Ginsenoside Rg3-S | SIRT2 |
| Ginsenoside Rd | PSEN2 | Ginsenoside Rd | ADAM17 | Ginsenoside Rg3-R | PPARG | Ginsenoside Rg3-S | F2 |
| Ginsenoside Rd | FGF2 | Ginsenoside Rd | IDE | Ginsenoside Rg3-R | F2 | Ginsenoside Rg3-S | AR |
| Ginsenoside Rd | IL2 | Ginsenoside Rg3-R | PSEN1 | Ginsenoside Rg3-R | AR | Ginsenoside Rg3-S | MMP1 |
| Ginsenoside Rd | HSD11B2 | Ginsenoside Rg3-R | PSEN2 | Ginsenoside Rg3-R | MME | Ginsenoside Rg3-S | MME |
| Ginsenoside Rd | SLC5A2 | Ginsenoside Rg3-R | FGF2 | Ginsenoside Rg3-R | MTOR | Ginsenoside Rg3-S | MTOR |
| Ginsenoside Rd | SLC5A1 | Ginsenoside Rg3-R | HTR2B | Ginsenoside Rg3-R | PIK3CA | Ginsenoside Rg3-S | PIK3CA |
| Ginsenoside Rd | LGALS3 | Ginsenoside Rg3-R | ADRA2A | Ginsenoside Rg3-S | PSEN1 | Ginsenoside Rg3-S | PIK3CG |
| Ginsenoside Rd | IGF1R | Ginsenoside Rg3-R | DRD1 | Ginsenoside Rg3-S | PSEN2 | Ginsenoside Rg3-S | EGFR |
| Ginsenoside Rd | XIAP | Ginsenoside Rg3-R | HTR2C | Ginsenoside Rg3-S | FGF2 | Ginsenoside Rg3-S | MAPK8 |
| Ginsenoside Rd | CASP3 | Ginsenoside Rg3-R | CYP2D6 | Ginsenoside Rg3-S | CYP2D6 | Ginsenoside Rg3-S | MAPK14 |
| Ginsenoside Rd | P2RY12 | Ginsenoside Rg3-R | IL2 | Ginsenoside Rg3-S | IL2 | Ginsenoside Rg3-S | AXL |
| Ginsenoside Rd | PTPN22 | Ginsenoside Rg3-R | HSD11B2 | Ginsenoside Rg3-S | HSD11B2 | Ginsenoside Rg3-S | MERTK |
| Ginsenoside Rd | ITGB3 | Ginsenoside Rg3-R | SLC5A2 | Ginsenoside Rg3-S | SLC5A2 | Ginsenoside Rg3-S | CXCR2 |
| Ginsenoside Rd | REN | Ginsenoside Rg3-R | SLC5A1 | Ginsenoside Rg3-S | SLC5A1 | Ginsenoside Rg3-S | HTR1A |
| Ginsenoside Rd | LIPC | Ginsenoside Rg3-R | LGALS3 | Ginsenoside Rg3-S | LGALS3 | Ginsenoside Rg5 | FGF2 |
| Ginsenoside Rd | MMP2 | Ginsenoside Rg3-R | XIAP | Ginsenoside Rg3-S | IGF1R | Ginsenoside Rg5 | IL2 |
| Ginsenoside Rd | SRC | Ginsenoside Rg3-R | CASP3 | Ginsenoside Rg3-S | CASP3 | Ginsenoside Rg5 | HSD11B2 |
| Ginsenoside Rd | ACE | Ginsenoside Rg3-R | PTPN22 | Ginsenoside Rg3-S | PTPN22 | Ginsenoside Rg5 | SLC5A2 |
| Ginsenoside Rd | F7 | Ginsenoside Rg3-R | ITGB3 | Ginsenoside Rg3-S | ITGB3 | Ginsenoside Rg5 | SLC5A1 |
| Ginsenoside Rd | DPP4 | Ginsenoside Rg3-R | REN | Ginsenoside Rg3-S | REN | Ginsenoside Rg5 | IGF1R |
| Ginsenoside Rd | PTPN1 | Ginsenoside Rg3-R | SLC6A2 | Ginsenoside Rg3-S | MMP2 | Ginsenoside Rg5 | CASP3 |
| Ginsenoside Rd | DHFR | Ginsenoside Rg3-R | MMP2 | Ginsenoside Rg3-S | ACE | Ginsenoside Rg5 | P2RY12 |
| Ginsenoside Rg5 | PTPN22 | Ginsenoside Rk1 | ADRA2A | Ginsenoside Rk1 | ADRB3 | Gypenoside J1 | LIPC |
| Ginsenoside Rg5 | ITGB3 | Ginsenoside Rk1 | CYP2D6 | Ginsenoside Rk1 | DGAT1 | Gypenoside J1 | MMP2 |
| Ginsenoside Rg5 | REN | Ginsenoside Rk1 | IL2 | Ginsenoside Rk1 | NR4A2 | Gypenoside J1 | SRC |
| Ginsenoside Rg5 | LIPC | Ginsenoside Rk1 | HSD11B2 | Ginsenoside Rk1 | ADAM17 | Gypenoside J1 | ACE |
| Ginsenoside Rg5 | MMP2 | Ginsenoside Rk1 | SLC5A2 | Ginsenoside Rk1 | HTR1A | Gypenoside J1 | AGTR1 |
| Ginsenoside Rg5 | F7 | Ginsenoside Rk1 | SLC5A1 | Gypenoside J1 | PSEN1 | Gypenoside J1 | DRD2 |
| Ginsenoside Rg5 | PTPN1 | Ginsenoside Rk1 | CASP3 | Gypenoside J1 | PSEN2 | Gypenoside J1 | DRD3 |
| Ginsenoside Rg5 | PPARG | Ginsenoside Rk1 | P2RY12 | Gypenoside J1 | FGF2 | Gypenoside J1 | HRAS |
| Ginsenoside Rg5 | ADRB2 | Ginsenoside Rk1 | REN | Gypenoside J1 | HTR2B | Gypenoside J1 | F7 |
| Ginsenoside Rg5 | ALDH2 | Ginsenoside Rk1 | MMP2 | Gypenoside J1 | ADRA2A | Gypenoside J1 | DPP4 |
| Ginsenoside Rg5 | MME | Ginsenoside Rk1 | ACE | Gypenoside J1 | DRD1 | Gypenoside J1 | AKR1B1 |
| Ginsenoside Rg5 | MTOR | Ginsenoside Rk1 | F7 | Gypenoside J1 | HTR2C | Gypenoside J2 | PSEN1 |
| Ginsenoside Rg5 | PIK3CA | Ginsenoside Rk1 | PTPN1 | Gypenoside J1 | CYP2D6 | Gypenoside J2 | PSEN2 |
| Ginsenoside Rg5 | PIK3CG | Ginsenoside Rk1 | ADRB2 | Gypenoside J1 | IL2 | Gypenoside J2 | IL2 |
| Ginsenoside Rg5 | EGFR | Ginsenoside Rk1 | SIRT2 | Gypenoside J1 | HSD11B2 | Gypenoside J2 | SLC5A2 |
| Ginsenoside Rg5 | MAPK14 | Ginsenoside Rk1 | MMP1 | Gypenoside J1 | SLC5A2 | Gypenoside J2 | SLC5A1 |
| Ginsenoside Rg5 | F9 | Ginsenoside Rk1 | MME | Gypenoside J1 | SLC5A1 | Gypenoside J2 | IGF1R |
| Ginsenoside Rg5 | CXCR2 | Ginsenoside Rk1 | MTOR | Gypenoside J1 | LGALS3 | Gypenoside J2 | XIAP |
| Ginsenoside Rg5 | GBA2 | Ginsenoside Rk1 | PIK3CG | Gypenoside J1 | IGF1R | Gypenoside J2 | CASP3 |
| Ginsenoside Rg5 | UGCG | Ginsenoside Rk1 | EGFR | Gypenoside J1 | XIAP | Gypenoside J2 | P2RY12 |
| Ginsenoside Rg5 | ADRB3 | Ginsenoside Rk1 | MAPK8 | Gypenoside J1 | CASP3 | Gypenoside J2 | PTPN22 |
| Ginsenoside Rg5 | JAK2 | Ginsenoside Rk1 | MAPK14 | Gypenoside J1 | P2RY12 | Gypenoside J2 | ITGB3 |
| Ginsenoside Rk1 | PSEN1 | Ginsenoside Rk1 | CXCR2 | Gypenoside J1 | PTPN22 | Gypenoside J2 | REN |
| Ginsenoside Rk1 | PSEN2 | Ginsenoside Rk1 | MAP2K1 | Gypenoside J1 | ITGB3 | Gypenoside J2 | MMP2 |
| Ginsenoside Rk1 | FGF2 | Ginsenoside Rk1 | GBA2 | Gypenoside J1 | REN | Gypenoside J2 | SRC |
| Ginsenoside Rk1 | HTR2B | Ginsenoside Rk1 | UGCG | Gypenoside J1 | SLC6A2 | Gypenoside J2 | ACE |
| Gypenoside J2 | F7 | Gypenoside L | LGALS3 | Gypenoside LI | SLC5A2 | Gypenoside LI | MERTK |
| Gypenoside J2 | DPP4 | Gypenoside L | IGF1R | Gypenoside LI | SLC5A1 | Gypenoside LVI | PSEN1 |
| Gypenoside J2 | AKR1B1 | Gypenoside L | XIAP | Gypenoside LI | LGALS3 | Gypenoside LVI | PSEN2 |
| Gypenoside J2 | PTPN1 | Gypenoside L | CASP3 | Gypenoside LI | CASP3 | Gypenoside LVI | FGF2 |
| Gypenoside J2 | PPP2CA | Gypenoside L | PTPN22 | Gypenoside LI | P2RY12 | Gypenoside LVI | HTR2B |
| Gypenoside J2 | PPARG | Gypenoside L | ITGB3 | Gypenoside LI | PTPN22 | Gypenoside LVI | ADRA2A |
| Gypenoside J2 | ADRB2 | Gypenoside L | MMP2 | Gypenoside LI | ITGB3 | Gypenoside LVI | DRD1 |
| Gypenoside J2 | AKT2 | Gypenoside L | SRC | Gypenoside LI | REN | Gypenoside LVI | HTR2C |
| Gypenoside J2 | RPS6KA1 | Gypenoside L | ACE | Gypenoside LI | MMP2 | Gypenoside LVI | CYP2D6 |
| Gypenoside J2 | AKT1 | Gypenoside L | AGTR1 | Gypenoside LI | SRC | Gypenoside LVI | IL2 |
| Gypenoside J2 | ALDH2 | Gypenoside L | F7 | Gypenoside LI | ACE | Gypenoside LVI | HSD11B2 |
| Gypenoside J2 | F3 | Gypenoside L | AKR1B1 | Gypenoside LI | F7 | Gypenoside LVI | SLC5A2 |
| Gypenoside J2 | DHFR | Gypenoside L | PTPN1 | Gypenoside LI | AKR1B1 | Gypenoside LVI | SLC5A1 |
| Gypenoside J2 | SIRT2 | Gypenoside L | PPARG | Gypenoside LI | PTPN1 | Gypenoside LVI | LGALS3 |
| Gypenoside L | PSEN1 | Gypenoside L | AR | Gypenoside LI | PPARG | Gypenoside LVI | IGF1R |
| Gypenoside L | PSEN2 | Gypenoside L | MME | Gypenoside LI | F2 | Gypenoside LVI | CASP3 |
| Gypenoside L | FGF2 | Gypenoside L | MTOR | Gypenoside LI | HSD11B1 | Gypenoside LVI | P2RY12 |
| Gypenoside L | HTR2B | Gypenoside L | PIK3CA | Gypenoside LI | AR | Gypenoside LVI | PTPN22 |
| Gypenoside L | ADRA2A | Gypenoside L | PIK3CG | Gypenoside LI | MMP1 | Gypenoside LVI | ITGB3 |
| Gypenoside L | DRD1 | Gypenoside LI | PSEN1 | Gypenoside LI | MME | Gypenoside LVI | REN |
| Gypenoside L | HTR2C | Gypenoside LI | PSEN2 | Gypenoside LI | PIK3CG | Gypenoside LVI | MMP2 |
| Gypenoside L | CYP2D6 | Gypenoside LI | FGF2 | Gypenoside LI | EGFR | Gypenoside LVI | SRC |
| Gypenoside L | IL2 | Gypenoside LI | HTR2C | Gypenoside LI | MAPK8 | Gypenoside LVI | DRD2 |
| Gypenoside L | HSD11B2 | Gypenoside LI | CYP2D6 | Gypenoside LI | INSR | Gypenoside LVI | DRD3 |
| Gypenoside L | SLC5A2 | Gypenoside LI | IL2 | Gypenoside LI | MAPK14 | Gypenoside LVI | F7 |
| Gypenoside L | SLC5A1 | Gypenoside LI | HSD11B2 | Gypenoside LI | AXL | Gypenoside LVI | PTPN1 |
| Gypenoside LVI | ADRB2 | Gypenoside XLVI | PSEN2 | Gypenoside XLVI | PTPN22 | Gypenoside XLVI | DHFR |
| Gypenoside LVI | AKT2 | Gypenoside XLVI | FGF2 | Gypenoside XLVI | ITGB3 | Gypenoside XLVI | AR |
| Gypenoside LVI | RPS6KA1 | Gypenoside XLVI | IL2 | Gypenoside XLVI | REN | Gypenoside XLVI | NR3C1 |
| Gypenoside LVI | AKT1 | Gypenoside XLVI | HSD11B2 | Gypenoside XLVI | LIPC | Gypenoside XLVI | MME |
| Gypenoside LVI | HSD11B1 | Gypenoside XLVI | SLC5A2 | Gypenoside XLVI | MMP2 | Gypenoside XLVI | PIK3CG |
| Gypenoside LVI | AR | Gypenoside XLVI | SLC5A1 | Gypenoside XLVI | SRC | Gypenoside XLVI | MAP2K1 |
| Gypenoside LVI | NR3C1 | Gypenoside XLVI | IGF1R | Gypenoside XLVI | ACE | Gypenoside XLVI | ADAM17 |
| Gypenoside LVI | INSR | Gypenoside XLVI | XIAP | Gypenoside XLVI | F7 | Gypenoside XLVI | IDE |
| Gypenoside LVI | FKBP5 | Gypenoside XLVI | CASP3 | Gypenoside XLVI | DPP4 |  |  |
| Gypenoside XLVI | PSEN1 | Gypenoside XLVI | P2RY12 | Gypenoside XLVI | PTPN1 |  |  |

**Table S3 PPI Network analysis of potential targets**

| **Name** | **Degree** | **Betweenness Centrality** | **Closeness Centrality** | **Neighborhood Connectivity** |
| --- | --- | --- | --- | --- |
| SRC | 22 | 0.28786434 | 0.56842105 | 8.31818182 |
| PIK3CA | 19 | 0.11730987 | 0.51923077 | 9.31578947 |
| HRAS | 17 | 0.13632757 | 0.52427184 | 9.29411765 |
| AKT1 | 16 | 0.08706325 | 0.47368421 | 8.875 |
| EGFR | 12 | 0.11284287 | 0.48214286 | 9.5 |
| JUN | 10 | 0.07184059 | 0.45378151 | 9.5 |
| AR | 10 | 0.02212481 | 0.45762712 | 12.3 |
| PPP2CA | 10 | 0.06670162 | 0.45 | 10.1 |
| MAPK14 | 9 | 0.02512253 | 0.432 | 9.77777778 |
| JAK2 | 9 | 0.09124576 | 0.45762712 | 11.55555556 |
| IL2 | 9 | 0.02819121 | 0.43548387 | 11 |
| MAP2K1 | 9 | 0.02514237 | 0.45378151 | 12 |
| MAPK8 | 8 | 0.05100149 | 0.43902439 | 10 |
| F2 | 8 | 0.153548 | 0.43902439 | 8 |
| MTOR | 8 | 0.05323939 | 0.421875 | 10 |
| PTPN1 | 8 | 0.00663703 | 0.45 | 12.5 |
| PRKCD | 8 | 0.02981702 | 0.43548387 | 11.25 |
| IGF1R | 8 | 0.0033701 | 0.43548387 | 12.125 |
| INSR | 7 | 0.00359934 | 0.41221374 | 12.14285714 |
| AKT2 | 7 | 0.00470511 | 0.39416058 | 10.85714286 |
| ITGB3 | 7 | 0.027883 | 0.432 | 11.57142857 |
| PIK3CG | 6 | 0.00519284 | 0.41221374 | 12.83333333 |
| NR3C1 | 6 | 0.00374495 | 0.38848921 | 10 |
| IGFBP3 | 5 | 0.02621445 | 0.38848921 | 7 |
| FGF2 | 5 | 0.01453756 | 0.38571429 | 7.2 |
| FGFR2 | 5 | 0.00673494 | 0.41860465 | 14.6 |
| AGTR1 | 4 | 0.07393628 | 0.33962264 | 4 |
| CASP3 | 4 | 0.00360055 | 0.35761589 | 8.75 |
| FKBP5 | 4 | 0.0009074 | 0.35526316 | 10 |
| MMP2 | 4 | 0.01165806 | 0.38028169 | 8.75 |
| ADRB2 | 3 | 0.00857085 | 0.38571429 | 10.66666667 |
| SERPINE1 | 3 | 0.01956285 | 0.35064935 | 6.33333333 |
| LGALS3 | 3 | 0.03703704 | 0.37241379 | 10 |
| F3 | 3 | 0.07267645 | 0.31395349 | 4 |
| MMP1 | 3 | 0.00049058 | 0.31395349 | 4.66666667 |
| UGCG | 3 | 0.03703704 | 0.36 | 9.66666667 |
| PPARG | 3 | 0.07285115 | 0.34394904 | 6.66666667 |
| ACE | 2 | 0 | 0.25592417 | 3 |
| REN | 2 | 0 | 0.25592417 | 3 |
| ADAM17 | 2 | 0.00085775 | 0.3483871 | 10.5 |
| HTR2C | 2 | 0 | 0.30857143 | 5.5 |
| XIAP | 2 | 0 | 0.32727273 | 10 |
| HSD11B1 | 2 | 0.03703704 | 0.25961538 | 2 |
| DPP4 | 2 | 1 | 1 | 1 |
| F9 | 2 | 0 | 0.24107143 | 2.5 |
| F7 | 2 | 0 | 0.24107143 | 2.5 |
| PIN1 | 2 | 0.00034941 | 0.33540373 | 9 |
| ABCB1 | 1 | 0 | 0.30681818 | 8 |
| AKR1B1 | 1 | 0 | 1 | 1 |
| DHFR | 1 | 0 | 1 | 1 |
| ALOX5 | 1 | 0 | 1 | 1 |
| PTGS2 | 1 | 0 | 1 | 1 |
| AXL | 1 | 0 | 0.36486486 | 22 |
| CXCR2 | 1 | 0 | 0.31213873 | 10 |
| CYP2D6 | 1 | 0 | 0.20689655 | 2 |
| SLC5A2 | 1 | 0 | 0.66666667 | 2 |
| MME | 1 | 0 | 0.66666667 | 2 |
| SLC5A1 | 1 | 0 | 0.32727273 | 12 |
| GBA2 | 1 | 0 | 0.26600985 | 3 |
| ICAM1 | 1 | 0 | 1 | 1 |
| SELE | 1 | 0 | 1 | 1 |
| MERTK | 1 | 0 | 0.27272727 | 3 |
| PTPN22 | 1 | 0 | 0.36486486 | 22 |
| TRPV4 | 1 | 0 | 0.36486486 | 22 |

**Table S4 GO enrichment analysis of the predicted targets for gypenosides treatment of GMD (Top 10)**

| **ID** | **Go term** | **Classification** | **Count** | **GeneRatio** | ***p* value** |
| --- | --- | --- | --- | --- | --- |
| GO:0003018 | vascular process in circulatory system | Biological process | 17 | 17/87 | 1.46E^-17^ |
| GO:0018108 | peptidyl-tyrosine phosphorylation | Biological process | 20 | 20/87 | 3.75E^-16^ |
| GO:0018212 | peptidyl-tyrosine modification | Biological process | 20 | 20/87 | 4.37E^-16^ |
| GO:0043491 | protein kinase B signaling | Biological process | 18 | 18/87 | 4.78E^-16^ |
| GO:0062197 | cellular response to chemical stress | Biological process | 19 | 19/87 | 2.87E^-15^ |
| GO:0043434 | response to peptide hormone | Biological process | 20 | 20/87 | 1.12E^-14^ |
| GO:0042493 | response to drug | Biological process | 19 | 19/87 | 1.69E^-14^ |
| GO:0043405 | regulation of MAP kinase activity | Biological process | 18 | 18/87 | 1.77E^-14^ |
| GO:0051896 | regulation of protein kinase B signaling | Biological process | 16 | 16/87 | 3.45E^-14^ |
| GO:0035296 | regulation of tube diameter | Biological process | 13 | 13/87 | 9.92E^-14^ |
| GO:0045121 | membrane raft | Cellular component | 17 | 17/87 | 7.80E^-14^ |
| GO:0098857 | membrane microdomain | Cellular component | 17 | 17/87 | 8.20E^-14^ |
| GO:0098589 | membrane region | Cellular component | 17 | 17/87 | 1.54E^-13^ |
| GO:0045177 | apical part of cell | Cellular component | 13 | 13/87 | 5.93E^-08^ |
| GO:0005901 | caveola | Cellular component | 7 | 7/87 | 7.82E^-08^ |
| GO:0031253 | cell projection membrane | Cellular component | 11 | 11/87 | 3.61E^-07^ |
| GO:0016324 | apical plasma membrane | Cellular component | 11 | 11/87 | 5.82E^-07^ |
| GO:0044853 | plasma membrane raft | Cellular component | 7 | 7/87 | 7.11E^-07^ |
| GO:0098978 | glutamatergic synapse | Cellular component | 10 | 10/87 | 4.59E^-06^ |
| GO:0005911 | cell-cell junction | Cellular component | 11 | 11/87 | 1.16E^-05^ |
| GO:1901338 | catecholamine binding | Molecular function | 6 | 6/87 | 2.77E^-11^ |
| GO:0043560 | insulin receptor substrate binding | Molecular function | 5 | 5/87 | 5.27E^-10^ |
| GO:0004713 | protein tyrosine kinase activity | Molecular function | 10 | 10/87 | 8.20E^-10^ |
| GO:0008144 | drug binding | Molecular function | 9 | 9/87 | 1.51E^-09^ |
| GO:0004175 | endopeptidase activity | Molecular function | 15 | 15/87 | 2.13E^-09^ |
| GO:0008227 | G protein-coupled amine receptor activity | Molecular function | 6 | 6/87 | 1.45E^-07^ |
| GO:0031994 | insulin-like growth factor I binding | Molecular function | 4 | 4/87 | 3.26E^-07^ |
| GO:0004714 | transmembrane receptor protein tyrosine kinase activity | Molecular function | 6 | 6/87 | 4.29E^-07^ |
| GO:0001618 | virus receptor activity | Molecular function | 6 | 6/87 | 1.36E^-06^ |
| GO:0140272 | exogenous protein binding | Molecular function | 6 | 6/87 | 1.36E^-06^ |

**Table S5 KEGG pathways enrichment analysis of potential targets for gypenosides treatment of GMD (Top 20)**

| **ID** | **Term** | **Gene** | **Count** | **GeneRatio** | ***p* value** |
| --- | --- | --- | --- | --- | --- |
| hsa04933 | AGE-RAGE signaling pathway in diabetic complications | CASP3, MMP2, AGTR1, HRAS, AKT2, AKT1, F3, JUN, PRKCD | 16 | 16/83 | 2.05E^-15^ |
|  |  | PIK3CA, MAPK8, MAPK14, ICAM1, SELE, SERPINE1, JAK2 |  |  |  |
| hsa01522 | Endocrine resistance | CYP2D6, IGF1R, MMP2, SRC, HRAS, AKT2, AKT1, JUN | 15 | 15/83 | 3.34E^-14^ |
|  |  | ABCB11, MTOR, PIK3CA, EGFR, MAPK8, MAPK14, MAP2K1 |  |  |  |
| hsa01521 | EGFR tyrosine kinase inhibitor resistance | FGF2, IGF1R, SRC, HRAS, AKT2, AKT1, MTOR | 13 | 13/83 | 7.47E^-13^ |
|  |  | PIK3CA, EGFR, AXL, MAP2K1, FGFR2, JAK2 |  |  |  |
| hsa05167 | Kaposi sarcoma-associated herpesvirus infection | FGF2, CASP3, SRC, HRAS, AKT2, AKT1, JUN, PTGS2, MTOR | 16 | 16/83 | 7.41E^-11^ |
|  |  | PIK3CA, PIK3CG, MAPK8, MAPK14, ICAM1, MAP2K1, JAK2 |  |  |  |
| hsa05207 | Chemical carcinogenesis - receptor activation | FGF2, XIAP, SRC, HRAS, ADRB2, AKT2, RPS6KA1, AKT1 | 16 | 16/83 | 2.81E^-10^ |
|  |  | JUN, AR, MTOR, PIK3CA, EGFR, MAP2K1, ADRB3, JAK2 |  |  |  |
| hsa04722 | Neurotrophin signaling pathway | PSEN1, PSEN2, HRAS, AKT2, RPS6KA1, AKT1, JUN | 12 | 12/83 | 2.19E^-09^ |
|  |  | PRKCD, PIK3CA, MAPK8, MAPK14, MAP2K1 |  |  |  |
| hsa04926 | Relaxin signaling pathway | MMP2, SRC, HRAS, AKT2, AKT1, JUN, MMP1 | 12 | 12/83 | 5.57E^-09^ |
|  |  | PIK3CA, EGFR, MAPK8, MAPK14, MAP2K1 |  |  |  |
| hsa04625 | C-type lectin receptor signaling pathway | IL2, SRC, HRAS, AKT2, AKT1, JUN, PRKCD | 11 | 11/83 | 6.49E^-09^ |
|  |  | PTGS2, PIK3CA, MAPK8, MAPK14 |  |  |  |
| hsa04066 | HIF-1 signaling pathway | IGF1R, AKT2, AKT1, SLC2A1, PFKFB3, MTOR | 11 | 11/83 | 1.07E^-08^ |
|  |  | PIK3CA, EGFR, INSR, MAP2K1, SERPINE1 |  |  |  |
| hsa04012 | ErbB signaling pathway | SRC, HRAS, AKT2, AKT1, JUN, MTOR, | 10 | 10/83 | 1.17E^-08^ |
|  |  | PIK3CA, EGFR, MAPK8, MAP2K1 |  |  |  |
| hsa05418 | Fluid shear stress and atherosclerosis | ITGB3, MMP2, SRC, AKT2, AKT1, JUN, TRPV4, | 12 | 12/83 | 1.31E^-08^ |
|  |  | PIK3CA, MAPK8, MAPK14, ICAM1, SELE |  |  |  |
| hsa05210 | Colorectal cancer | CASP3, HRAS, AKT2, AKT1, JUN, | 10 | 10/83 | 1.32E^-08^ |
|  |  | MTOR, PIK3CA, EGFR, MAPK8, MAP2K1 |  |  |  |
| hsa04668 | TNF signaling pathway | CASP3, AKT2, AKT1, JUN, PTGS2, PIK3CA, | 11 | 11/83 | 1.43E^-08^ |
|  |  | MAPK8, MAPK14, ICAM1, SELE, MAP2K1 |  |  |  |
| hsa05205 | Proteoglycans in cancer | FGF2, IGF1R, CASP3, ITGB3, MMP2, SRC, HRAS, AKT2, | 14 | 14/83 | 1.51E^-08^ |
|  |  | AKT1, MTOR, PIK3CA, EGFR, MAPK14, MAP2K1 |  |  |  |
| hsa05235 | PD-L1 expression and PD-1 checkpoint pathway in cancer | HRAS, AKT2, AKT1, JUN, MTOR, PIK3CA, | 10 | 10/83 | 1.84E^-08^ |
|  |  | EGFR, MAPK14, MAP2K1, JAK2 |  |  |  |
| hsa04015 | Rap1 signaling pathway | FGF2, IGF1R, ITGB3, SRC, DRD2, HRAS, AKT2, AKT1, | 14 | 14/83 | 2.06E^-08^ |
|  |  | PIK3CA, EGFR, INSR, MAPK14, MAP2K1, FGFR2 |  |  |  |
| hsa04072 | Phospholipase D signaling pathway | AGTR1, HRAS, AKT2, AKT1, F2, MTOR, | 12 | 12/83 | 2.66E^-08^ |
|  |  | PIK3CA, PIK3CG, EGFR, INSR, CXCR2, MAP2K1 |  |  |  |
| hsa05417 | Lipid and atherosclerosis | CASP3, SRC, HRAS, PPARG, AKT2, AKT1, JUN, MMP1, | 14 | 14/83 | 2.78E^-08^ |
|  |  | PIK3CA, MAPK8, MAPK14, ICAM1, SELE, JAK2 |  |  |  |
| hsa04917 | Prolactin signaling pathway | SRC, HRAS, AKT2, AKT1, PIK3CA, | 9 | 9/83 | 3.00E^-08^ |
|  |  | MAPK8, MAPK14, MAP2K1, JAK2 |  |  |  |


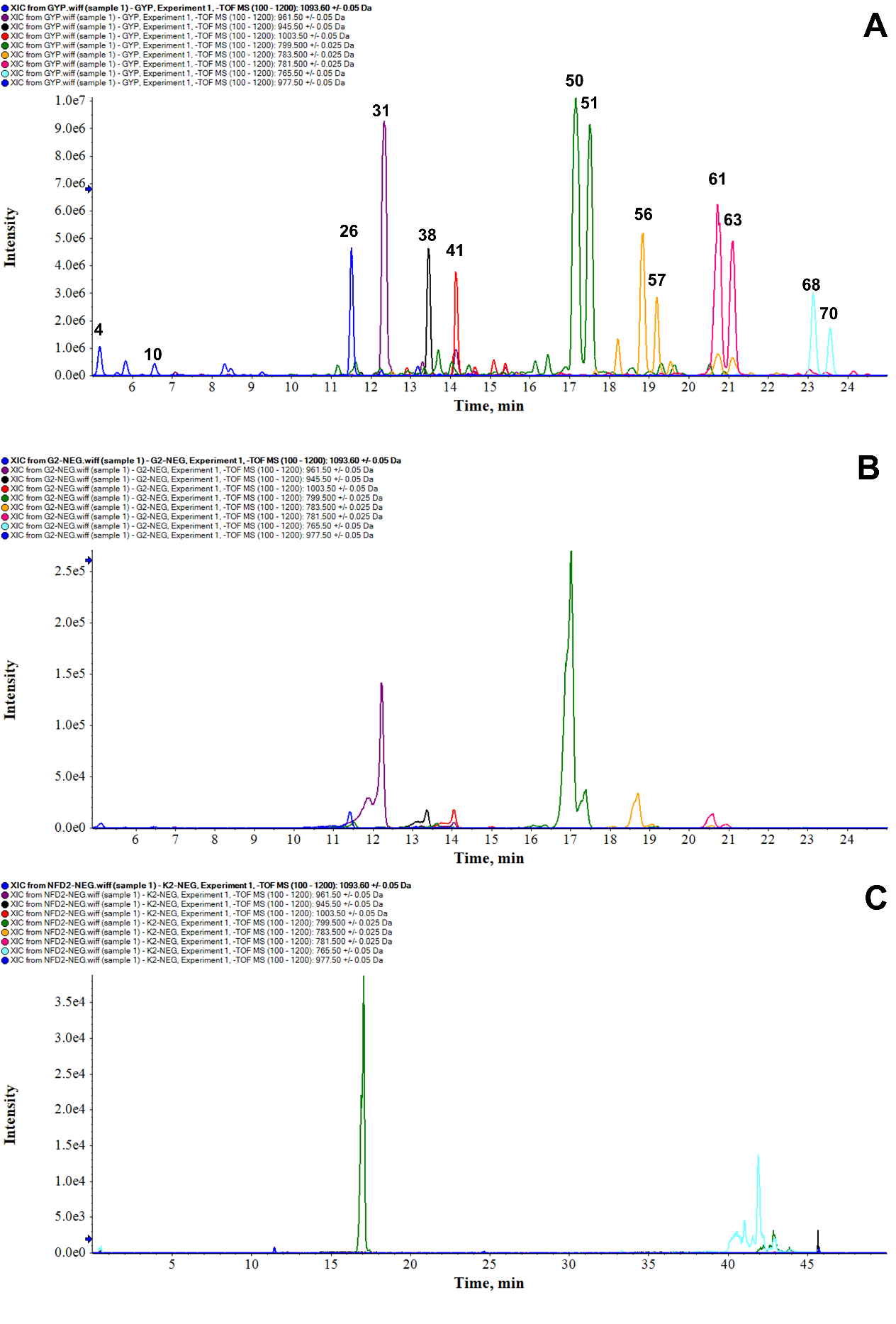


**Figure S1.** XIC of 14 serum components. (A) HGyp; (B) Serum of HGyp; (C) Blank serum.
